# Identification of Vaccine-Reactive *Campylobacter jejuni* Outer Membrane Proteins Recognized by Antibodies from Immunized Layer Breeders and Their Offspring

**DOI:** 10.3390/vaccines14100850

**Published:** 2026-09-27

**Authors:** Mostafa Naguib, Mohamed A. Abouelkhair, Ali Nazmi, Khaled Abdelaziz

**Affiliations:** 1 Department of Animal and Veterinary Science, Clemson University, Clemson, SC 29634, USA; mnaguib@clemson.edu; 2 Department of Poultry Diseases, Faculty of Veterinary Medicine, Cairo University, Cairo 12211, Egypt; 3 Department of Veterinary Biomedical Sciences, Rowan University, Glassboro, NJ 08062, USA; abouelkhair@rowan.edu; 4 Department of Animal Sciences, The Ohio State University, Columbus, OH 43013, USA; nazmi.1@osu.edu; 5 Food for Health Discovery Theme, The Ohio State University, Columbus, OH 43013, USA; 6 Clemson University School of Health Research (CUSHR), Clemson, SC 29634, USA

**Keywords:** chicken, *Campylobacter*, vaccine, CpG ODN, outer membrane proteins

## Abstract

Background/Objectives: Our recent study demonstrated that vaccination of layer breeders with *Campylobacter jejuni* outer membrane proteins (OMPs) and CpG ODN increased serum IgY levels and enhanced maternal antibody transfer to their offspring. However, it remained unclear which specific proteins within the OMP fraction were responsible for these effects. Methods: In this study, Western blotting (WB) and ultra-high-performance liquid chromatography–tandem mass spectrometry (UHPLC–MS/MS) were used to identify proteins associated with elevated antibody responses. Results: Among the identified proteins, seven were found in high abundance and showed strong reactivity with both hen and offspring sera. These proteins included major outer membrane protein (MOMP), fibronectin type III domain protein (FlpA), type IV secretion system protein (VirB10), capsular polysaccharide ABC transporter (KpsD), flagellin (FlaA), flagellar hook protein (FlgE), and flagellar hook-associated protein (FliD). Conclusion: Several immunoreactive candidate proteins were identified in this study; however, further validation via recombinant expression is required to evaluate their potential as vaccine candidates against *C. jejuni* in chickens.

## 1. Introduction

*Campylobacter*, a Gram-negative, motile, spiral-shaped bacterium, poses a significant public health threat, causing 1.5 million infections annually in the US [1], with associated healthcare costs reaching up to 11 billion dollars [2]. Animal-derived products, particularly poultry and their products, account for 50–80% of human infections, with *Campylobacter jejuni* being the most frequently isolated species [3]. Although this bacterium colonizes the chicken cecum at high densities of up to 10^9^ CFU/g of cecal content without causing clinical disease in chickens, the consumption of contaminated poultry products is frequently associated with gastroenteritis in humans, particularly among immunocompromised individuals, the elderly, and children [4]. Therefore, reducing *Campylobacter* burden in chickens is a crucial approach for preventing transmission to humans. Extensive research evaluated the efficacy of various vaccination strategies against *C. jejuni* in chickens, including killed, live attenuated, subunit, recombinant, and nanovaccine approaches [5]. However, despite these efforts, no commercial vaccine is currently available to control *C. jejuni* in chickens.

We have recently demonstrated that vaccination of layer breeders with a crude mixture of *C. jejuni* OMPs increased serum IgY antibody levels by approximately threefold and resulted in sustained high levels of maternally derived antibodies (MDA) in their offspring [6]. However, it remains unclear which immunogenic protein(s) within this mixture were responsible for the observed immune responses. Identifying these proteins may facilitate the development of an effective single- or multi-antigen vaccine capable of eliciting robust immune responses to control *C. jejuni.* Using Western blotting and ultra-high-performance liquid chromatography–tandem mass spectrometry (UHPLC–MS/MS), this study aimed to identify proteins associated with elevated antibody responses in the sera of hens and their offspring following administration of a crude *C. jejuni* OMPs preparation.

## 2. Materials and Methods

### 2.1. Preparation of C. jejuni Culture

A pure culture of *C. jejuni* strain 81-176 was prepared as previously described [7] (for details, see Supplementary Materials).

### 2.2. Preparation of C. jejuni-OMPs

*C. jejuni*-OMPs were extracted following the same protocol previously described [7] (for details, see Supplementary Materials).

### 2.3. CpG-ODN

A synthetic class B 2007 CpG oligodeoxynucleotide (ODN) with a fully phosphorothioate backbone was purchased from Invivogen (San Diego, CA, USA).

### 2.4. Experimental Design

Forty breeders (100-week-old white leghorns) and 4 roosters were assigned to four groups (A–D), with ten breeders and a rooster per group. All groups were vaccinated subcutaneously as follows: group A (200 μg *C. jejuni* OMPs), group B (50 μg CpG-ODN), group C (200 μg *C. jejuni* OMPs and 50 μg CpG-ODN), and group D (PBS), with a booster vaccination dose received three weeks later. While antibody levels were measured in our previous study from one to four weeks post-vaccination in breeders and from one to five weeks of age in hatched chickens, serum samples corresponding to peak antibody responses (four weeks in breeders (n = 10 serum samples) and four weeks of age in hatched chicks (n = 5 serum samples)) were considered for this study.

### 2.5. Enzyme-Linked Immunosorbent Assay (ELISA) for Measuring Campylobacter-Specific Antibody Levels in Hens’ and Chicks’ Sera

IgY antibody levels were measured as previously described [8] (for details, see Supplementary Materials).

### 2.6. SDS-PAGE and Immunoblot Analysis

A sodium dodecyl sulfate-polyacrylamide gel electrophoresis (SDS-PAGE) using 8% polyacrylamide gel was used to separate the OMPs of *C. jejuni* following the same procedure previously described by Laemmli [9] (for details, see Supplementary Materials). The band’s intensity was analyzed using Bio-Rad Image Lab software version 6.1.0.07 and then normalized with the intensity of the corresponding ladder band.

### 2.7. UHPLC–MS/MS Analysis

UHPLC–MS/MS was used to identify immunogenic proteins detectable by Western blotting. The OMPs extracted from *C. jejuni* were separated by SDS-PAGE and stained with CBB-R250. Individual protein bands were carefully excised, targeting only those in alignment with the reactive bands observed in the corresponding immunoblot. Afterward, the excised bands were subjected to trypsin digestion using the Pierce™ In-Gel Tryptic Digestion Kit (Thermo Fisher Scientific, Bartlett, IL, USA), followed by UHPLC-MS/MS (Thermo Scientific Orbitrap Fusion Tribrid mass spectrometer) (for more details, see Supplementary Materials).

### 2.8. Statistical Analysis

The data were analyzed using JMP^®^ Pro 19.0.3 (901023). The Shapiro–Wilk test was used to check the normality of the antibody levels data. Given that the data were non-normally distributed, antibody levels were analyzed using the Kruskal–Wallis test followed by Dunn’s test. *p* values less than 0.05 (*p* < 0.05) are considered significant for all tests. Pearson’s r correlation coefficient was used to evaluate the relationship between IgY antibody levels and the intensity of reactive bands.

## 3. Results

### 3.1. Identification of Cross-Reactive Antigens by Immunoblot Analysis

#### 3.1.1. Reactivity of Sera from Vaccinated Hens with Different Vaccine Formulations Against *C. jejuni* OMPs of the Homologous Strain

While ELISA results describing the kinetic antibody response were previously published, we used data from a single time point, corresponding to peak antibody levels, from both breeders and their offspring to correlate with band intensity observed in Western blotting. Briefly, for breeders, the ELISA results revealed significantly higher IgY levels in groups that received OMPs alone or OMPs combined with CpG ODN than in the control group (*p* < 0.0041, *p* < 0.0001, respectively) (Figure 1). For offspring, IgY MDA levels in offspring whose parents received the combination of OMPs and CpG ODN were significantly higher (*p* < 0.01) than in the control group at 4 weeks post-hatch (Figure 1). Despite the lower hatchability rate, likely due to the age of the hens used in this study, and the consequent small sample size of only five hatched birds, antibody levels remained significantly different from those of the unvaccinated control group.

To determine the reactivity of the antibodies contained in the serum samples, *C. jejuni* OMPs extracts were separated by SDS-PAGE, transferred to nitrocellulose membranes and immunoblotted. The reactivity of protein bands on the nitrocellulose membrane with sera from the group that received OMPs alone showed consistent banding profiles in 7 of 9 samples (Figure 2a). Similar patterns were also observed in the group that received OMPs combined with CpG ODN, with all nine samples showing comparable banding profiles (Figure 2c). The molecular weights of the reactive protein bands ranged from 45 to 90 kDa (Figure 2). Consistent reactivity was observed in most samples for the 90-, 60-, and 45 kDa proteins, whereas a few samples also reacted with the 69 kDa protein (Figure 2a,c). No reactivity was observed with the sera from CpG ODN alone or the PBS control (Figure 2b,d). The original immunoblot images are provided in the Supplementary Material (Figure S1). It is worth noting that the intensity of the band for the 90 kDa proteins was positively correlated with IgY antibody levels in the sera of vaccinated hens with the OMPs alone (*p* = 0.038) or OMPs combined with CpG ODN (*p* = 0.056) (Supplementary Figure S2).

#### 3.1.2. Reactivity of Sera from Hatched Layer Chicks (Offspring) of Corresponding Vaccinated Hen Groups Against *C. jejuni* OMPs of the Homologous Strain

Of five immunoblots per group, four chicks from parents that received the combination of OMPs and CpG ODN showed reactivity with sera similar to that observed in their parents (Figure 3c), whereas only two chicks from the parent group that received OMPs alone showed weak reactive bands (Figure 3a). As in their parents, no reactive bands were observed in the offspring groups from parents that received CpG ODN alone or the PBS control group (Figure 3b,d). The original immunoblots images are provided in the Supplementary Material (Figure S3). No correlation was observed between the intensities of the 45 and 60 kDa bands and IgY antibody levels in the group that received OMPs alone (Figures S4 and S5).

### 3.2. Identification of the Immune-Reactive Proteins by UHPLC–MS/MS

UHPLC-MS/MS was performed with the four bands of the OMP extract prepared from the *C. jejuni* 81-176 strain, which showed cross-reactivity with hens and offspring sera. The Uniprot proteome FASTA file UP000000646 was used as the search database for protein identification from UHPLC-MS/MS data. Over 90 proteins were identified after searching against the *C. jejuni* 81-176 database (total of 1748 sequences) (Supplementary Table S1). However, many of these identified proteins are localized to the cytoplasm, periplasm, or inner membrane. The identified proteins with high protein FDR confidence were then filtered based on the apparent molecular weight of the reactive bands, combined with the coverage percentage, abundance, Score Sequest HT and PSMs score. Four proteins were identified in band 4 (the 45 kDa protein), including the major outer membrane protein (MOMP), the fibronectin type III domain protein (FlpA), the type IV secretion system protein (VirB10), and an uncharacterized protein. Two proteins were identified in the 60 kDa band (band 3): the capsular polysaccharide ABC transporter (KpsD) and flagellin (FlaA). The 90 kDa protein (band 1) was identified as a flagellar hook protein (FlgE), while the 69 kDa protein (band 2) was identified as a flagellar hook-associated protein (FliD). The abundance of these proteins is depicted in Figure 4 (for complete characterization of the identified proteins, see Supplementary Tables S1–S3). The best-fit identified proteins for the four reactive bands are summarized in Table 1.

## 4. Discussion

The identification of highly immunogenic, conserved antigens for the development of cross-protective vaccines against *C. jejuni* in chickens remains a challenge. Evidence suggests that several highly conserved proteins are present in the outer membrane of *C. jejuni* [10]. As such, numerous vaccination studies have explored crude mixtures of *C. jejuni* OMPs as subunit vaccines to control *C. jejuni* infection in chickens, with promising outcomes [11]. Our earlier study showed that subcutaneous vaccination of either broilers or layer breeders with a vaccine containing *C. jejuni* OMPs and the toll-like receptor 21 ligand CpG ODN elicited high serum IgY antibody levels and reduced *Campylobacter* colonization [6,8]. However, the specific protein(s) responsible for eliciting immune responses and protective effects remain unclear.

While the separation of the OMP extract on the SDS-PAGE revealed around 20 protein bands, the immunoblots using sera from breeder hens at the peak of *C. jejuni*-OMP-specific antibodies (four weeks after vaccination) identified only four reactive bands with apparent molecular masses of 90, 69, 60 and 45 kDa. Obviously, these bands were identified in the groups that received the OMPs alone or in combination with CpG ODN. It was clear that the 90 kDa band was the most prominent reactive band. This consistently appeared in almost all tested sera from the group that received the OMP-CpG ODN combination and in 78% sera from the OMPs-alone group. However, a significant positive correlation between band intensity and IgY antibody levels was observed in the group that received OMPs alone.

Regarding the reactivity of the offspring’s serum samples, the group that received the combination of CpG ODN and OMPs exhibited a similar pattern of reactive bands to their parent, while only two birds showed a weak 90 kDa band in those from OMP-vaccinated parents. These findings expand upon and confirm our earlier data, indicating that CpG ODN, when combined with OMPs, elicits higher antibody responses in vaccinated hens and sustains MDA levels in their offspring [6].

We further sought to identify reactive proteins by excising the four bands from the SDS-PAGE gel corresponding to the reactive immunoblot bands, followed by UHPLC–MS/MS analysis. Despite careful excision of the four reactive bands before gel digestion, more than one protein fraction per band was detectable, making it difficult to determine which protein(s) were specifically recognized by the antibodies. Nonetheless, correlating protein abundance and molecular weight with immunoreactive bands on the membranes of hens and their offspring enabled us to identify potential candidate immunoreactive proteins warranting further investigation as candidates for single- or multi-antigen vaccines.

Despite advances in genome sequencing and web algorithms predicting the antigenic peptides recognized by CD4 cells, the successful identification of OMP candidate proteins remains a bottleneck [12]. The glycine extraction method is reliable and yields a high concentration of OMPs; however, a caveat is the presence of impurities, including cytoplasmic and periplasmic proteins [13], as observed in this study. Additionally, while earlier reports relied on data derived from the reference genome of *C. jejuni* NCTC 11168, the proteins identified in our study were based on the available Uniprot KB database for *C. jejuni* strain 81-176, the same strain used to prepare the OMP extract, which provides reliable protein annotations.

Flagellar hook protein (FlgE) and the flagellar filament protein FlaA, key factors in bacterial motility and gut colonization, are widely considered immunogenic. In this study, the 90 kDa reactive band was identified as FlgE, and the 60 kDa band was annotated as FlaA. These findings align with those of Shoaf-Sweeney et al. (2008), who observed a prominent reactive band at 89.4 and 59.7 kDa in an OMP extract probed with MDA sera from chicks from *C. jejuni* colonized parents; these bands were subsequently identified as FlgE and FlaA proteins, respectively [14].

A versatile pore-forming protein, the major outer membrane protein (MOMP), with a known molecular mass of 45 kDa, is essential for adhesion and interaction with host cells. This protein has previously been used as a recombinant subunit vaccine and demonstrated strong protection against heterologous *C. jejuni* strains in mice; however, its efficacy in chickens has not yet been investigated [15]. The fibronectin type III domain protein (FlpA) identified in this study is a crucial adhesin, alongside CadF, that binds to host cells and activates host-cell signaling [16]. In an intestinally focused reverse vaccinology study, FlgE, FlpA, MOMP/PorA, and FlaA were identified as highly conserved proteins across multiple *C. jejuni* strains, underscoring their potential as novel vaccine candidates against heterologous *C. jejuni* strains [17]. However, it is important to note that not all immunogenic proteins confer protective immunity. As such, while the high intensity observed for the 90 kDa protein (FlgE) suggests strong immunogenic potential, its protective and neutralizing capacities require further investigation.

Type IV secretion system homolog (Cjp3/VirB10) genes were detected by sequence analysis, and N-linked glycosylation is required for their full competence [18]. Nevertheless, its potential as a candidate vaccine antigen is yet to be determined. Another identified antigen is the capsule polysaccharide (CPS), which has been investigated as a vaccine antigen against *C. jejuni* but has shown limited effectiveness. A key virulence component, *O*-methyl phosphoramidate (MeOPN), present in 75% of *C. jejuni* CPSs, modulates host innate immune response and cytokine release from mouse dendritic cells. The subcutaneous administration of a capsular polysaccharide-based vaccine conjugated with diphtheria toxoid, either with or without CpG or Addavax™ as adjuvant, at 3, 4, and 5 weeks old in SPF chickens elicited systemic IgY (up to 75% seroconversion) and reduced *C. jejuni* cecal count by 0.64 log_10_ at 38 days old [19]. However, commercial broilers received two doses of the same vaccine formulation and did not seroconvert before the *C. jejuni* challenge at 29 days old [19]. Although it showed limited efficacy in chickens, a CPS-based vaccine delivered SC in Aotus nancymaae monkeys provided 100% protection following challenge with wild *C. jejuni* 81-176 [20]. Candidate conserved immunoreactive proteins, including FlpA, flagellar proteins, and KpsD, would support the development of a cross-reactive multiantigen vaccine. On the other hand, variable surface regions, phase variation, and plasmid-restricted VirB10 challenge broad coverage and necessitate validation across heterologous strains.

## 5. Conclusions

In conclusion, this study identifies several candidate immunoreactive *C. jejuni* OMPs that show strong reactivity in the sera of vaccinated hens and their offspring. However, whether these proteins are targets of antibody binding and whether they exert protective effects remains elusive. Further validation of recombinant expression is required to establish these proteins as candidates for developing single- or multi-antigen vaccines to effectively control *C. jejuni* in chickens.

## Figures and Tables

**Figure 1:**
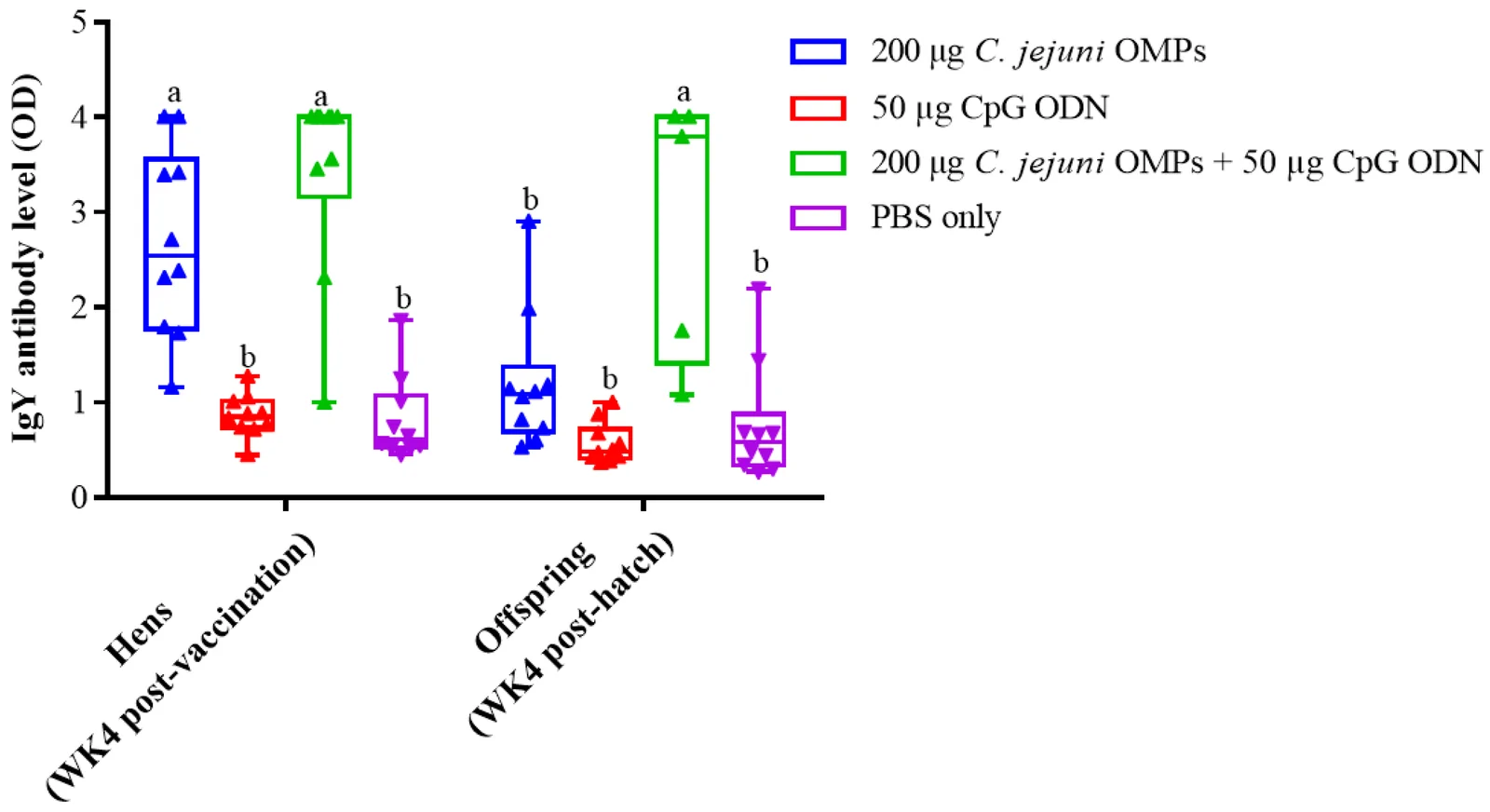
*C. jejuni*-specific IgY antibody levels in sera of vaccinated breeders and maternally derived antibodies of their offspring. Breeders were vaccinated SC with 200 µg of *C. jejuni* OMPs, 50 µg of CpG ODN, their combination or PBS. Booster doses were administered 3 weeks after primary vaccination. Blood samples were collected at 4 weeks post-primary vaccination and at 4 weeks post-hatch (offspring). Bars represent mean optical density (OD) ± standard error at 405 nm for nine to ten birds per group for breeders and five to ten birds per group for the offspring. Bars marked with the same letters (a, b) show no significant differences between groups, whereas those with different letters indicate significant differences (*p* < 0.05). OMPs = outer membrane proteins. CpG ODN = synthetic single-stranded oligodeoxynucleotides (ODNs) containing unmethylated CpG motifs.

**Figure 2:**
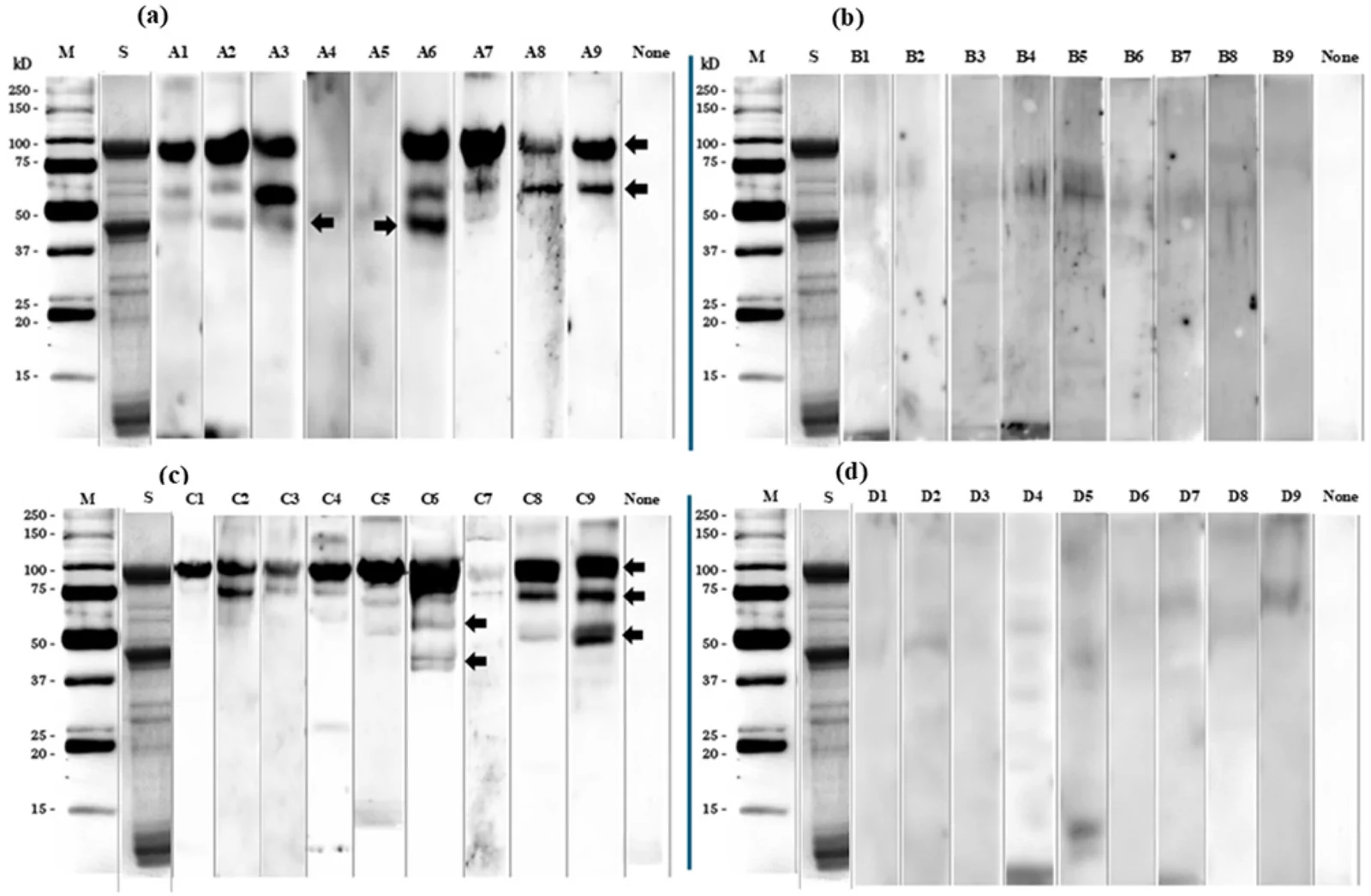
Immunoblots of the OMP extract of *C. jejuni* probed with hens’ sera. OMPs were prepared from *C. jejuni* (20 µg per well), separated by SDS-PAGE (S), and electroblotted onto nitrocellulose membranes. The breeders were vaccinated with either 200 µg of *C. jejuni* OMPs (**a**), or 50 µg of CpG ODN (**b**), or their combination (200 µg of *C. jejuni* OMPs and 50 µg of CpG ODN) (**c**), or PBS (**d**). At four weeks post-primary vaccination, breeders were bled, and nine serum samples per group were separated and probed on the strips A1–A9, B1–B9, C1–C9, and D1–D9. An empty well (no OMPs loaded) served as a control.

**Figure 3:**
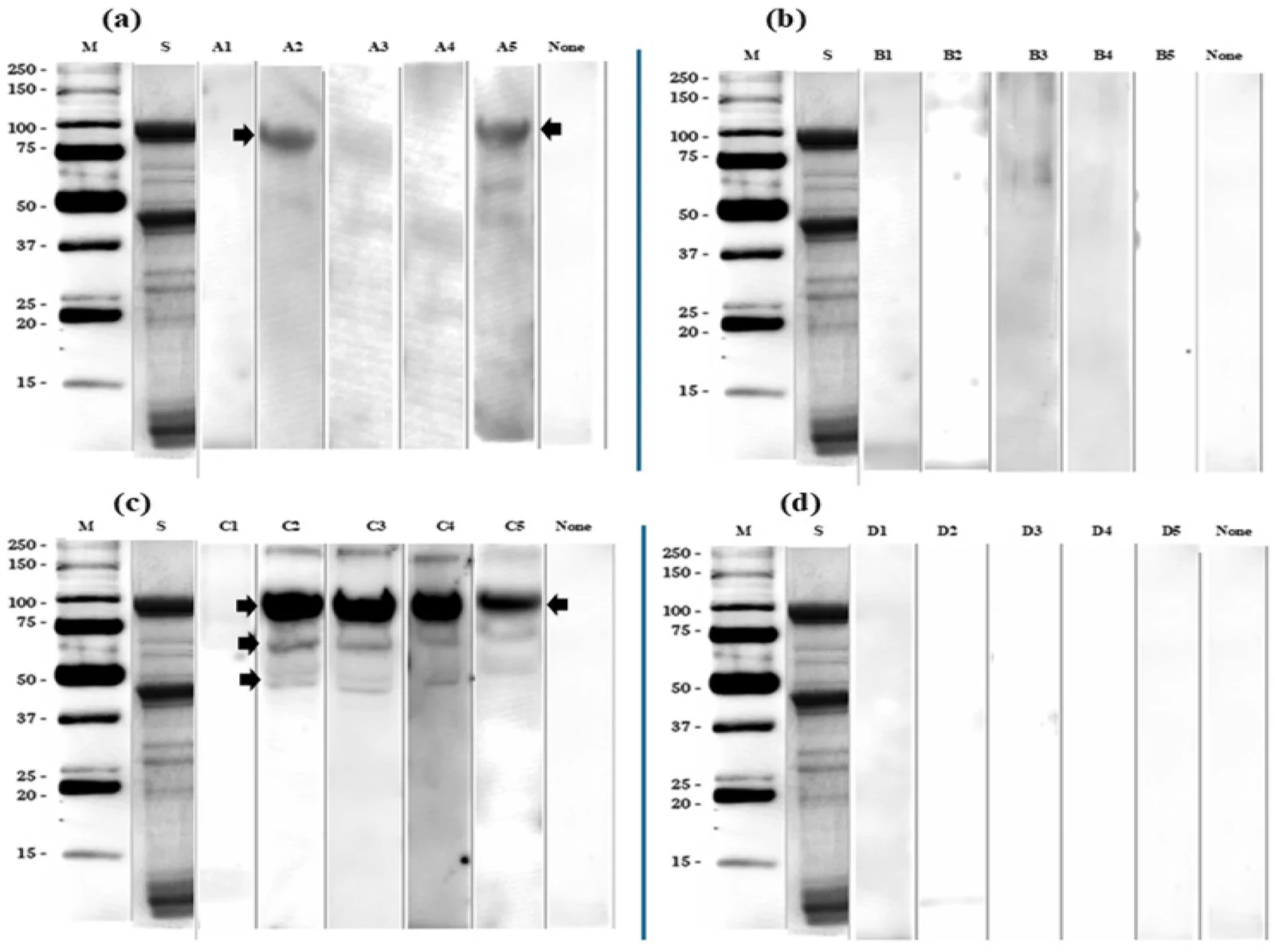
Immunoblots of the OMP extract of *C. jejuni* probed with the offspring’s sera. OMPs were prepared from *C. jejuni* (20 µg per well), separated by SDS-PAGE (S), and electroblotted onto nitrocellulose membranes. After four weeks post-hatch, chickens were bled, sera were subsequently separated, and the MDA were probed on the blots. Chickens in groups (**a**–**d**) are from parent hens vaccinated with either 200 µg of *C. jejuni* OMPs, 50 µg of CpG ODN, their combination or PBS, respectively. Black arrows point to the identified protein bands.

**Figure 4:**
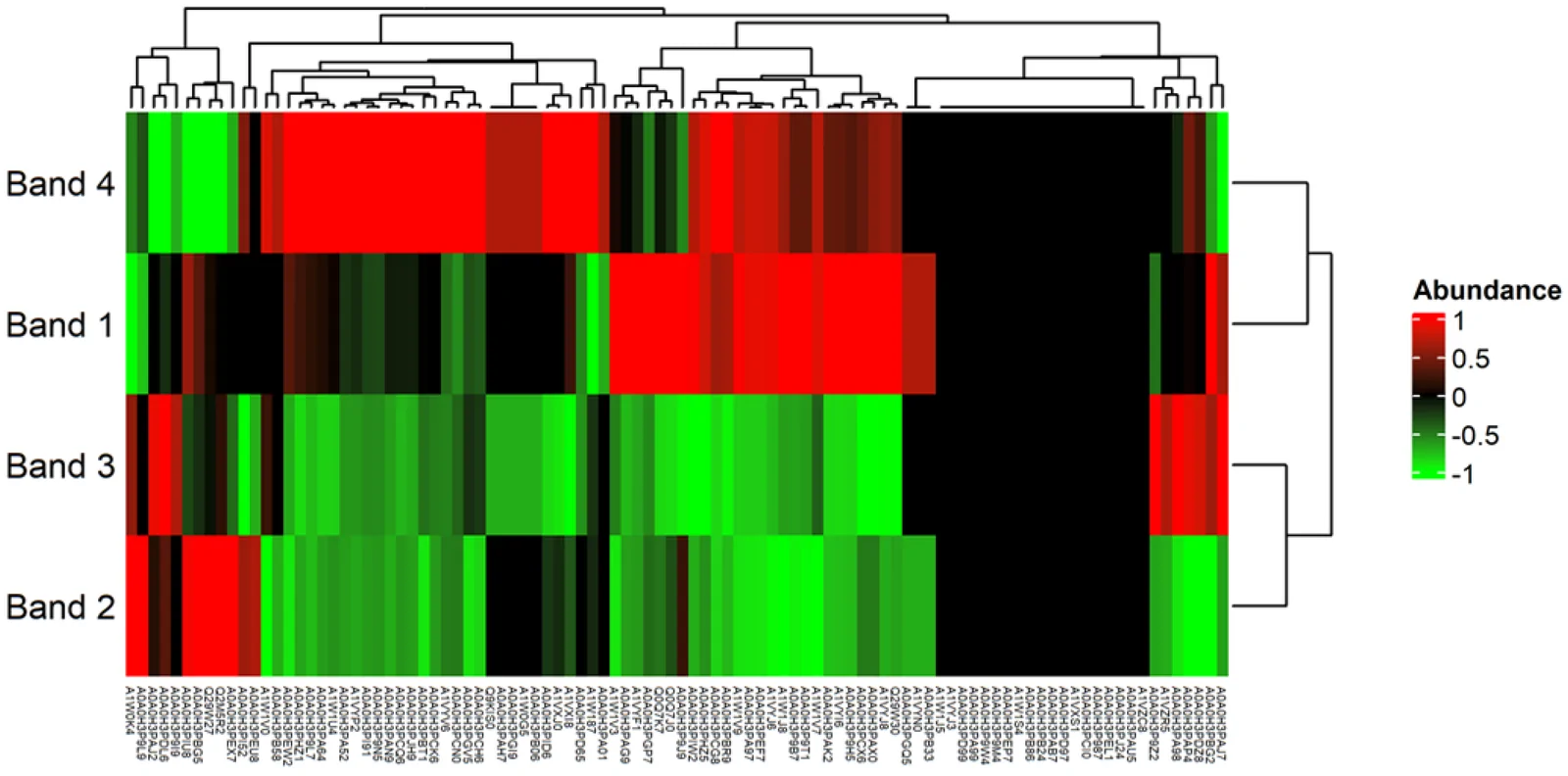
The abundance of the reactive proteins of the *C. jejuni*-OMP extract with the sera of vaccinated hens and their offspring was identified by UHPLC-MS/MS and analyzed by Uniprot KB database against the *C. jejuni* 81-176 strain. The heatmap was generated using R Core Team (version 4.4.1) software. The figure displays the non-normalized abundance values for each identified protein (Accession number) in the different bands.

**Table 1:** Immunoreactive OMPs with strong reactivity.

Band	Molecular Weight (kDa)	Protein	Protein Description	Coverage [%] ^a^	Score Sequest HT ^b^	PSMs ^c^	Cellular Localization	Role/Virulence
1	90	FlgE	Flagellar hook protein	31	94.41	25	Extracellular/Cell Surface	Motility
2	69	FliD	Flagellar hook-associated protein	33	97.3	29	Extracellular/Cell Surface	Attachment factor
3	60	KpsD	capsular polysaccharide ABC transporter	28	42.76	19	Outer membrane- associated	CPS Export protein
		FlaA	Flagellin	58	210.7	28	Extracellular/External Surface	Motility and cell invasion
4	45	MOMP/Por A	Major outer membrane protein	53	143.79	35	Outer Membrane	Pore-forming protein, adhesion and interaction with host cells
		FlpA	Fibronectin type III domain protein	11	13.32	4	Outer Membrane/Cell Surface	Fibronectin-like adhesin
		VirB10	Type IV secretion system protein	23	21.43	7	Trans-envelope complex	Competence

^a^ Percentage of the protein sequences covered by identified peptides. ^b^ Protein score, which is the sum of the scores of the individual peptides. ^c^ Number of identified peptide spectrum matches identified from all included searches.

## Data Availability

The data presented in this study are available on request from the corresponding author.

## Supplementary Materials

The following supporting information can be downloaded at: https://www.mdpi.com/article/10.3390/vaccines14100850/s1, Figure S1: The correlation between the intensity of the reactive 90-kDa band and the IgY antibody levels; Figure S2: Immunoblots of the OMPs extract of *C. jejuni* probed with hens’ sera; Figure S3: Immunoblots of the OMPs extract of *C. jejuni* probed with hens’ sera; Figure S4: The correlation between the intensity of the reactive 45-kDa band and the IgY antibody levels; Figure S5: The correlation between the intensity of the reactive 60-kDa band and the IgY antibody levels; Table S1: Major outer membrane protein OS = *Campylobacter jejuni* subsp. *jejuni* serotype O:23/36 (strain 81-176) OX = 354242 GN = porA PE = 4 SV = 1; Table S2: Characterization of immunogenic, membrane-associated proteins detected in reactive bands with sera from vaccinated hens and their offspring; Table S3: The abundance of the reactive proteins of the *C. jejuni*-OMPs extract with the sera of vaccinated hens and their offspring.
