# Supplementary material for: Identification of Vaccine-Reactive Campylobacter jejuni Outer Membrane Proteins Recognized by Antibodies from Immunized Layer Breeders and Their Offspring
Source: Vaccines (Basel). 2026 Sep 27;14(10):850. doi: 10.3390/vaccines14100850 (PMC13709648; doi:10.3390/vaccines14100850)
Supplement: Supplementary file 1 [file vaccines-14-00850-s001.zip › vaccines-4520760-Supplementary Files.pdf]

Material and Methods

Preparation of C. jejuni culture

A pure culture of *C. jejuni* strain 81-176 was prepared as previously described [1]. In brief, a frozen loopful of *C. jejuni* glycerol stock was streaked onto Brain Heart Infusion (BHI) agar supplemented with Preston selective *Campylobacter* supplement (Oxoid, Basingstoke, Hampshire, UK). The streaked plates were further incubated at 37°C for 48 hours in microaerophilic conditions (10% CO2, 5% O2, 85% N2). Afterward, several colonies were transferred to 5 mL of fresh BHI broth and incubated under the same microaerobic conditions for 48 hours at 37°C. Subsequently, 1 mL of this culture was inoculated into a 100 mL fresh BHI broth and re-incubated under the same conditions.

Preparation of *C. jejuni*-OMPs

C. jejuni-OMPs were extracted following the same protocol previously described [2]. Briefly, under the same abovementioned condition, *C. jejuni* was grown in 8 L of BHI broth and incubated for 40 hours. After centrifugation of the bacterial suspension at 3000× g for 10 min, the obtained pellet was washed twice with distilled water. Subsequently, 4 g of the pelleted cells were thoroughly mixed with 100 mL of 0.2 M glycine-hydrochloride buffer (pH 2.2), and the suspension was stirred at room temperature for 15 min. The suspension was centrifuged at 11,000× g for 15 min, and the supernatant was collected, neutralized with 1 N NaOH, and then dialyzed against deionized water overnight at 4 °C. The protein concentration in the OMPs suspension was measured by BCA Protein Assay Kit (Thermo Fisher Scientific, Rockford, IL, USA) and using SDS-PAGE protein separation, the bands were confirmed and visualized by Coomassie Blue staining (Figure 1). Subsequently, the OMPs suspension was stored at −80 °C until use.

Enzyme-Linked Immunosorbent Assay (ELISA) for Measuring Campylobacter-Specific Antibody Levels in hens' and chicks' sera

IgY antibody levels were measured as previously described [3]. In brief, Maxisorp 96-well plates (Thermo Fisher Scientific, Rochester, NY, USA) were coated with OMPs of *C. jejuni* (0.39 μg/100 μL) in PBS (pH 7.4) and incubated at 37°C for two hours. Afterward, plates were washed four times with PBS containing 0.05% Tween 20, followed by a blocking step with blocking buffer comprising 0.5% pig gelatin (Sigma, St. Louis, MO, USA) and 0.05% Tween 20 in PBS, and then incubated for one hour at 37°C. Hens' sera were diluted 1/1000, while offspring's sera samples were diluted (1/10) in a dilution buffer (PBS containing 1.5% Tween-20 and 0.29 M NaCl), and 100 μL was added to wells in duplicate. Subsequently, the plates were incubated at 37 °C for one hour, followed by four washes. Then, 100 μL of goat anti-chicken HRP-conjugated IgY (Sigma, USA) antibodies were added at dilutions of 1/4000. The secondary conjugated Ab were added to the wells, followed by an incubation at 37°C for 30 min. After washing with the washing buffer, 100 μL of ABTS substrate (Life Technologies, Frederick, MD, USA) was added to the wells, followed by another wash with the washing buffer. The plates were then incubated at room temperature for 30 min. To stop the reaction, 50 μL of 1% sodium dodecyl sulfate (Bio-Rad, Hercules, CA, USA) was added to the wells. The optical densities (OD) were measured at 405 nm.

SDS-PAGE and Immunoblot Analysis

A sodium dodecyl sulfate-polyacrylamide gel electrophoresis (SDS-PAGE) using 8% polyacrylamide gel was used to separate the OMPs of *C. jejuni* following the same procedure previously described by Laemmli [4] . Briefly, 20 μg of *C. jejuni* OMPs were solubilized in Laemmli buffer and heated to 100 °C for 5 minutes. The separated protein bands were either stained with Coomassie Blue R-250 or electroblotted onto a 0.22 μm supported nitrocellulose membrane (Bio-Rad, Germany). A phosphate-buffered saline with 5% non-fat milk and 0.05% (vol/vol) Tween-20 (PBS-T) was used to block the membrane. Avian serum samples were diluted into PBS-T as follows: hens' sera (1:1000), layer chicks' sera (1:50), and incubated for 1 h at room temperature with slight agitation. After washing three times with PBS-

T, the blots were incubated with goat anti-chicken HRP-conjugated IgY (Sigma, USA) at 1:4000 for 1 h at room temperature with slight agitation. After a final wash step, the blots were developed using Clarity Western ECL Substrate (Bio-Rad, California, USA) according to the manufacturer's instructions. The band's intensity was analyzed using Bio-Rad Image Lab software version 6.1.0.07 and then normalized with the intensity of the corresponding ladder band.

UHPLC–MS/MS analysis

UHPLC–MS/MS was used to identify immunogenic proteins detectable by Western blotting. The OMPs extracted from *C. jejuni* were separated by SDS-PAGE and stained with CBB-R250. Four reacted bands, 1, 2, 3 and 4, as depicted in Figure 1. Individual protein bands were carefully excised, targeting only those in alignment with the reactive bands observed in the corresponding immunoblot. Afterward, the excised bands were subjected to trypsin digestion using the Pierce™ In-Gel Tryptic Digestion Kit (Thermo Fisher Scientific, Illinois, USA), followed by UHPLC-MS/MS (Thermo Scientific Orbitrap Fusion Tribrid mass spectrometer). Briefly, Chromatographic separation was performed using a Thermo Scientific UltiMate 3000 RSLC nano system UHPLC coupled to Thermo Scientific Orbitrap Fusion Tribrid mass spectrometer with Thermo EASY SPRAY ESI source and PepMapTM RSLC C18 column (2 um, 100A, 75 um x 50 cm) under gradient conditions, using solvent A (0.1% formic acid in water, v/v) and solvent B (80% acetonitrile, 0.1% formic acid in water). The injection volume was set to 1 μL.

Mass spectrometry analyses were performed on an Orbitrap Fusion mass spectrometer (Thermo Fisher Scientific) equipped with an NSI Source. Source parameters were set as follows: spray voltage at +2200 V, ion transfer tube temperature at 275 °C. Chromatographic runs were analyzed over a total acquisition time of 170 min in positive ionization mode. MS1 scans were acquired in the Orbitrap analyzer at a resolution of 50,000 at m/z 200 with a scan range of m/z 300–1500, a maximum injection time of 50 ms, and an AGC target of 400000 (normalized to 100%). Microscans were set to 1, and data were recorded in profile mode. The data were analyzed using Proteome Discoverer (version 2.4.1.15) with the UniProtKB proteome FASTA database.

References

1. Taha-Abdelaziz, K.; Hodgins, D.C.; Alkie, T.N.; Quinteiro-Filho, W.; Yitbarek, A.; Astill, J.; Sharif, S. Oral administration of PLGA-encapsulated CpG ODN and Campylobacter jejuni lysate reduces cecal colonization by *Campylobacter jejuni* in chickens. *Vaccine* **2018**, *36*, 388–394.

2. Taha-Abdelaziz, K.; Hodgins, D.C.; Alkie, T.N.; Quinteiro-Filho, W.; Yitbarek, A.; Astill, J.; Sharif, S. Oral administration of PLGA-encapsulated CpG ODN and Campylobacter jejuni lysate reduces cecal colonization by *Campylobacter jejuni* in chickens. *Vaccine* **2018**, *36*, 388–394.

3. Hodgins, D.C.; Barjesteh, N.; St. Paul, M.; Ma, Z.; Monteiro, M.A.; Sharif, S. Evaluation of a polysaccharide conjugate vaccine to reduce colonization by *Campylobacter jejuni* in broiler chickens. *BMC Res. Notes* **2015**, *8*, 1–8.

4. Laemmli, U.K. Cleavage of structural proteins during the assembly of the head of bacteriophage T4. *Nature* **1970**, *227*, 680–685.

Supplemental Figures:

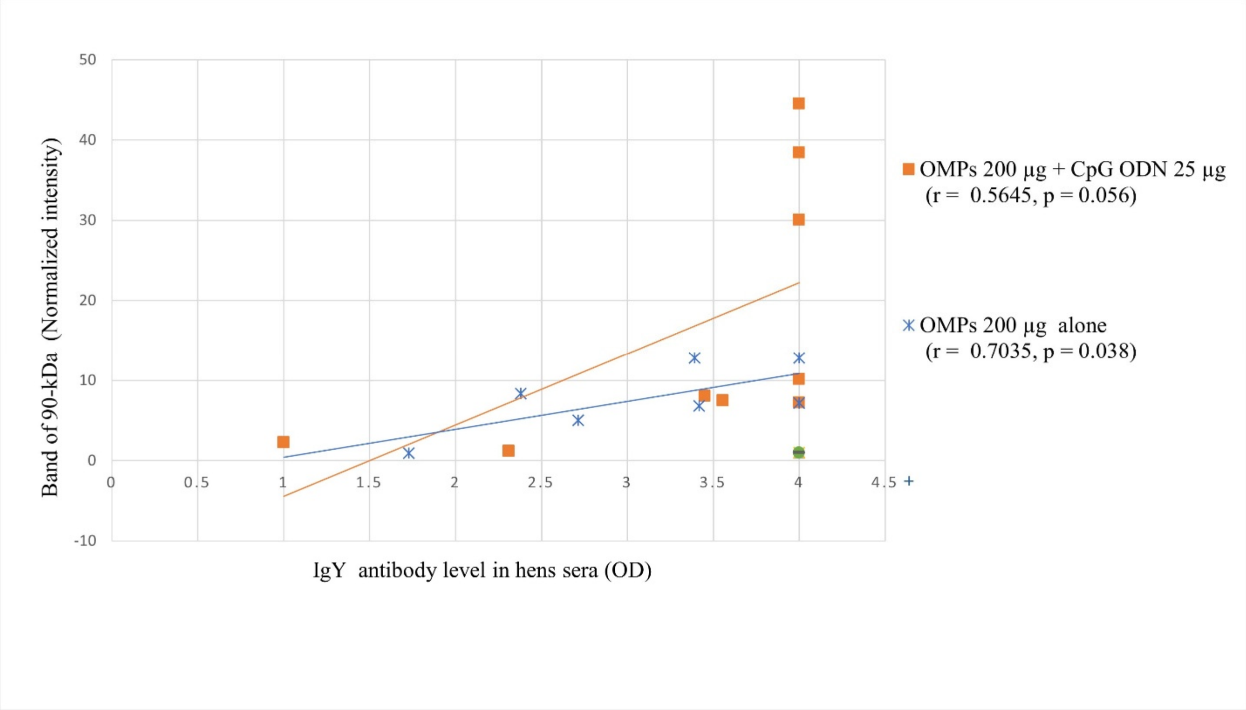

Figure S1. The correlation between the intensity of the reactive 90-kDa band and the IgY antibody levels in the sera of vaccinated hens was determined using Pearson's r correlation coefficient. A positive correlation was detected in the group that received OMPs alone (P=≤ 0.05).

Figure S2. Immunoblots of the OMPs extract of C. jejuni probed with hens' sera. OMPs were prepared from C. jejuni (20 µg per well), separated by SDS-PAGE (S), and electroblotted onto nitrocellulose membranes. The breeders were vaccinated with either 200 µg of C. jejuni OMPs (group A), or 50 µg of CpG ODN (group B), or their combination (200 µg of C. jejuni OMPs and 50 µg of CpG ODN) (group C), or PBS (group D). At four weeks post-primary vaccination, breeders were bled, and 9 serum samples per group were separated and probed on the strips A1-A9, B1-B9, C1-C9, and D1-D9. An empty well (no OMPs loaded) served as a control

1- Group A

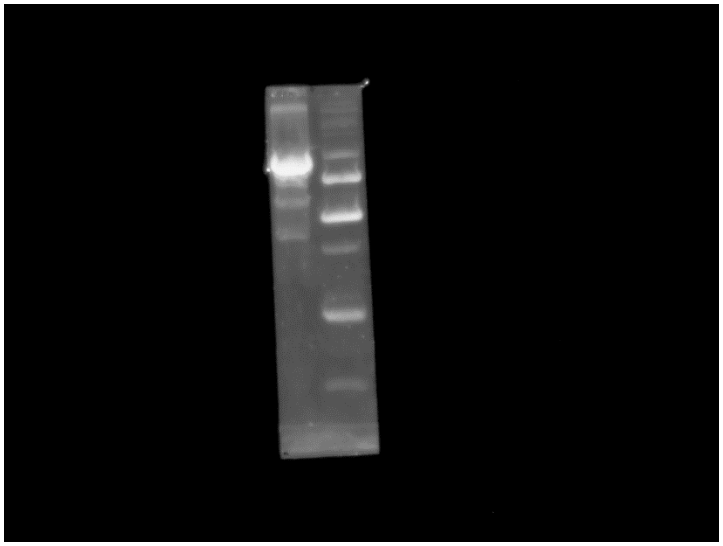

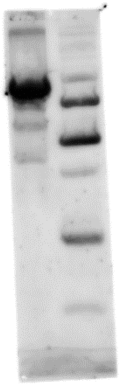

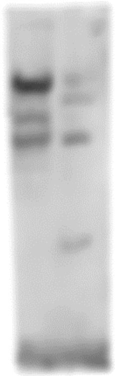

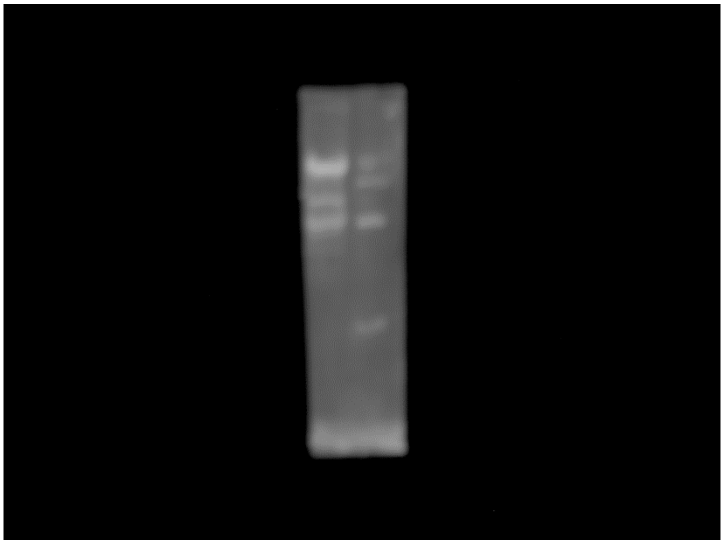

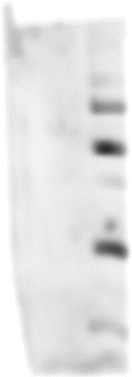

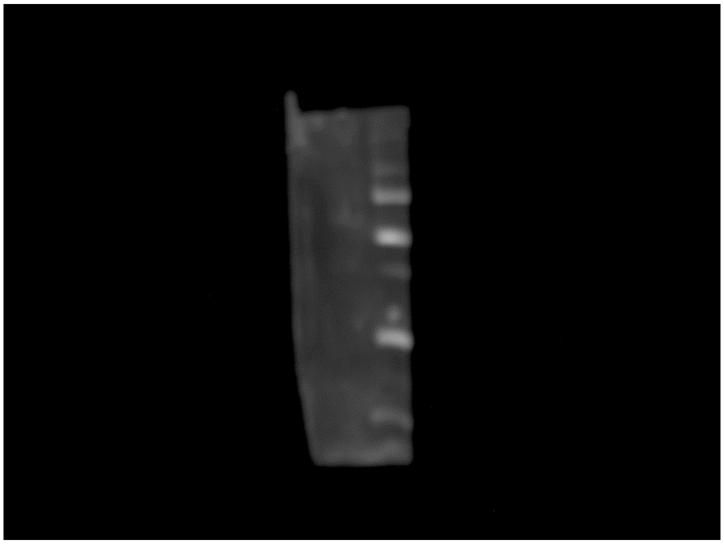

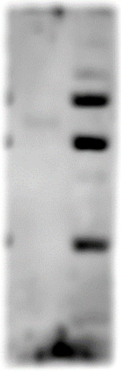

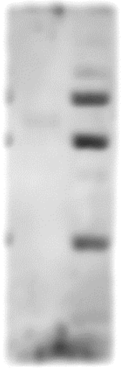

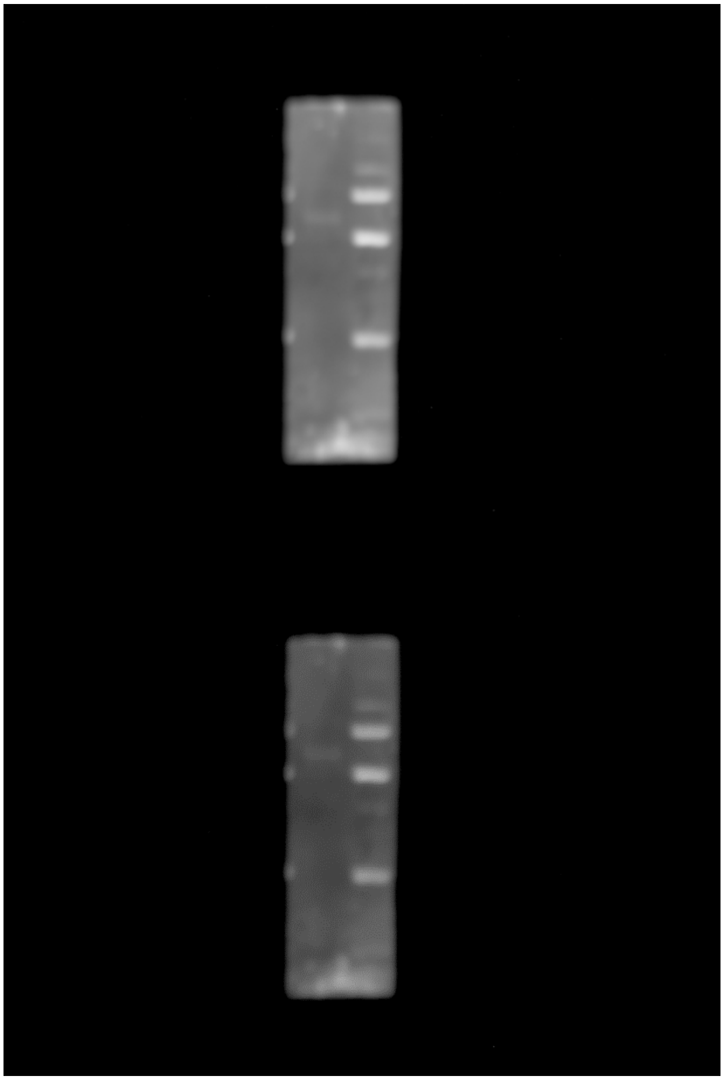

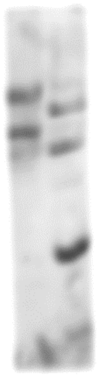

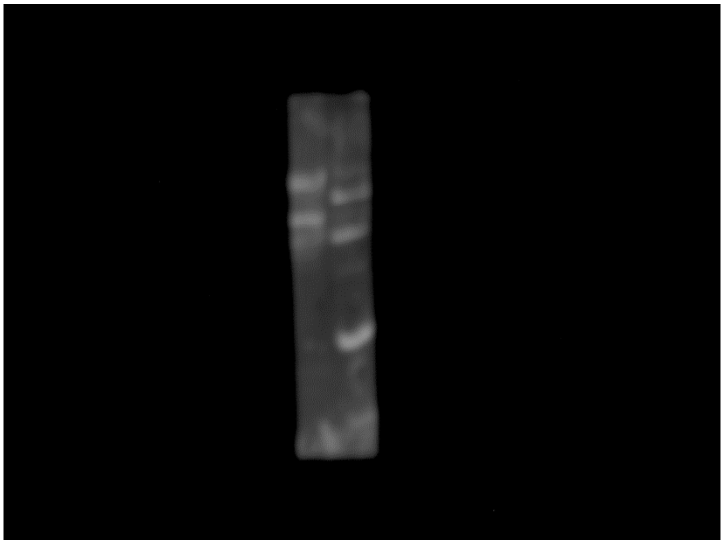

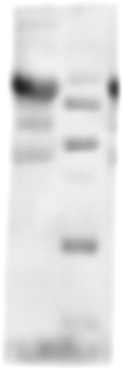

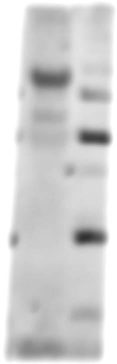
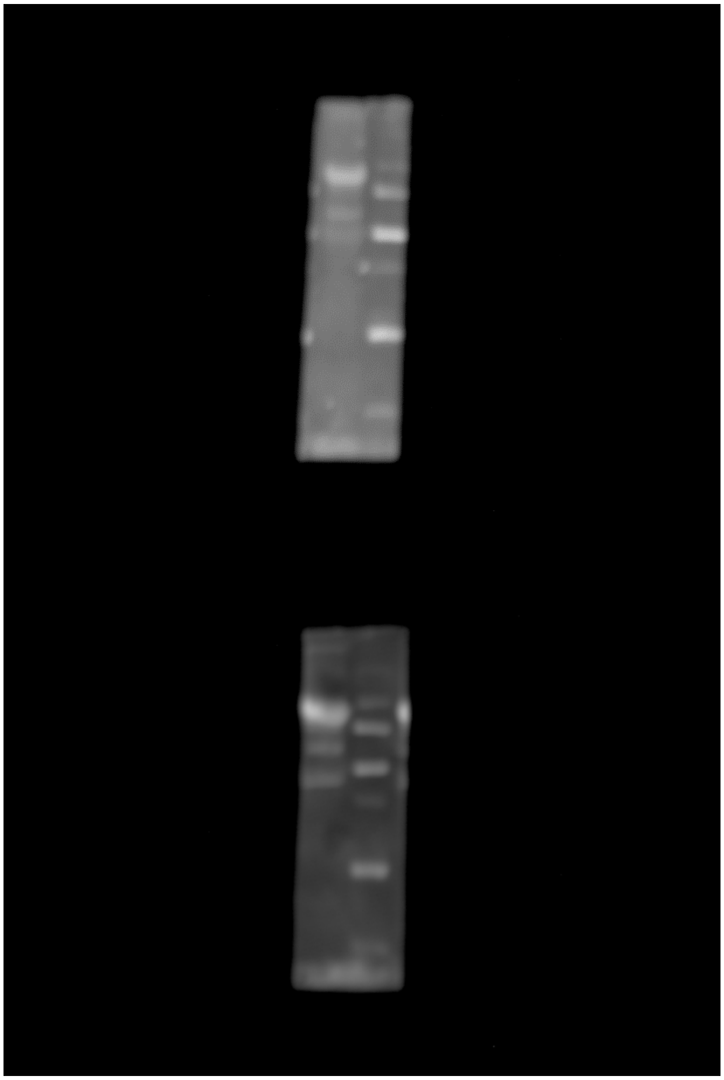

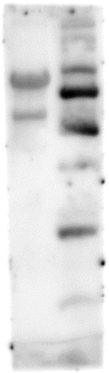
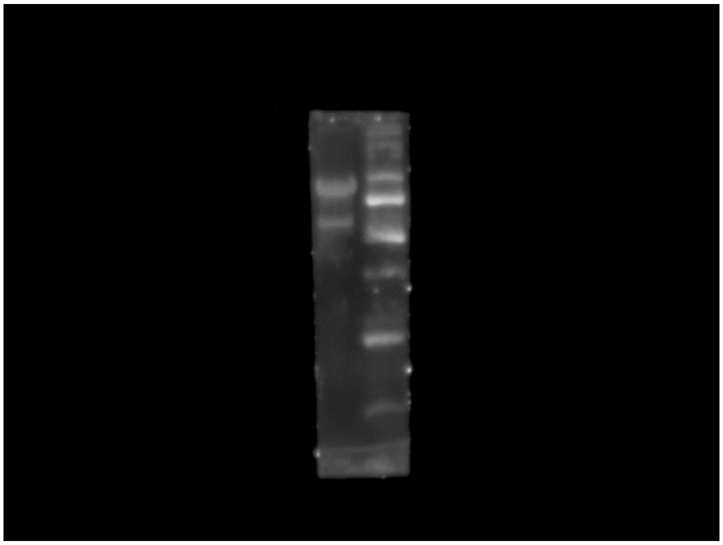
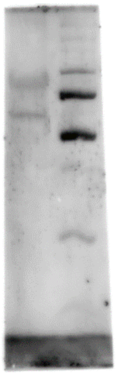

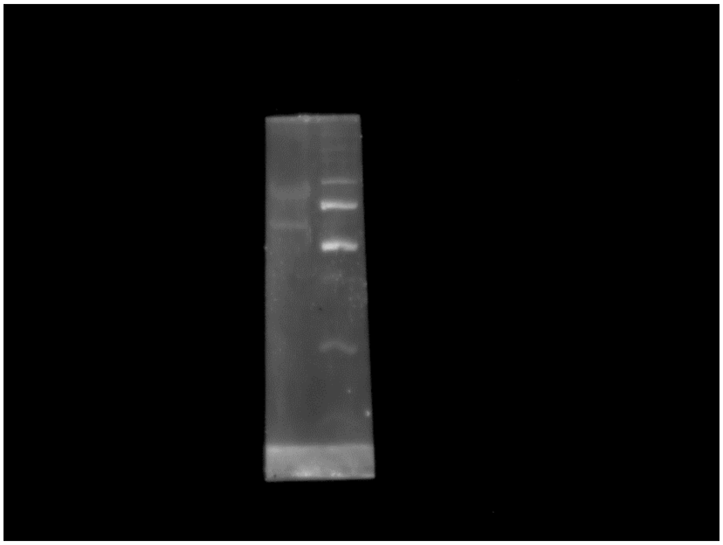

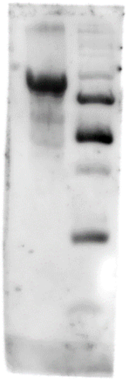

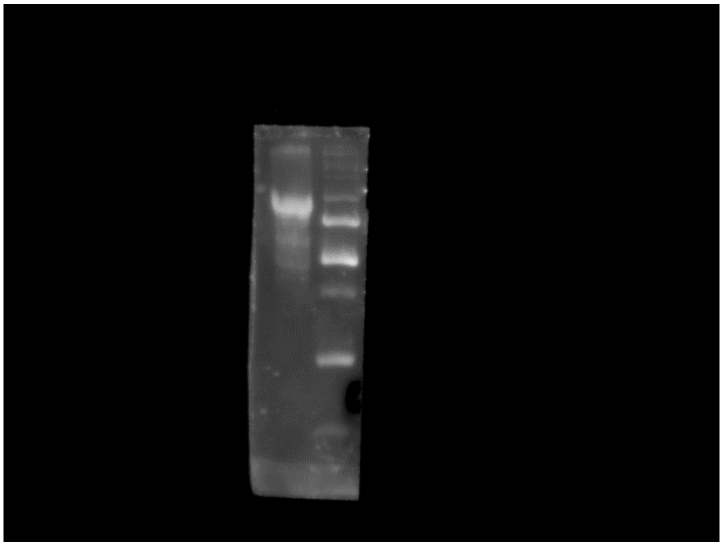

2- Group B

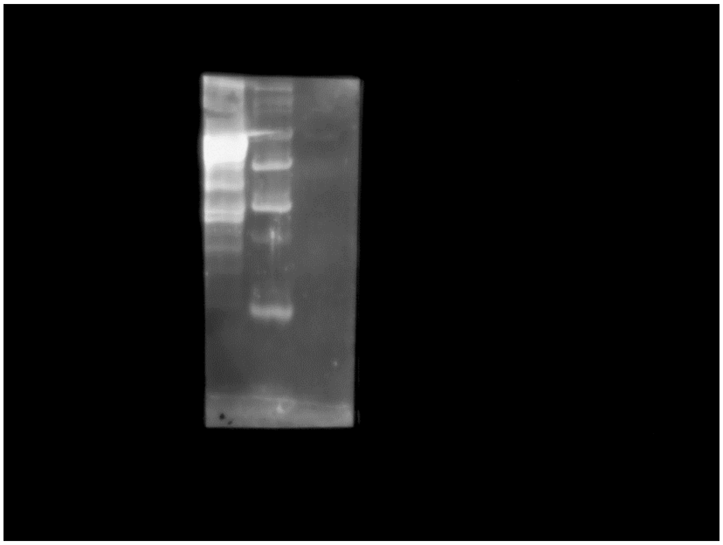

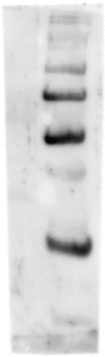

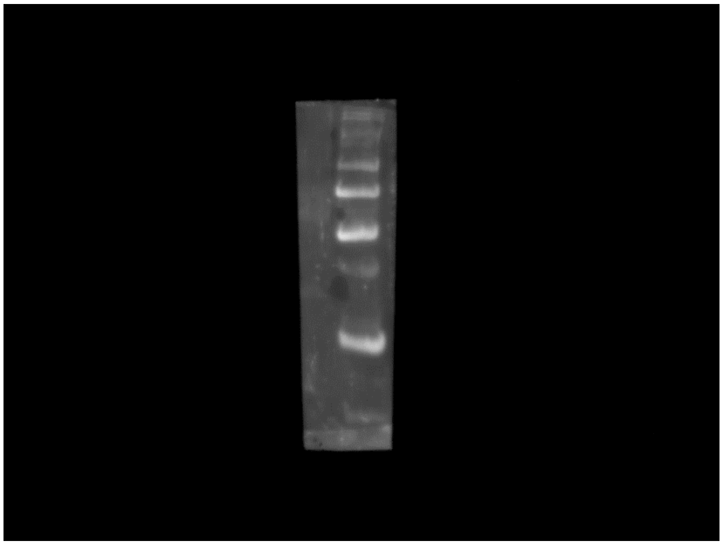

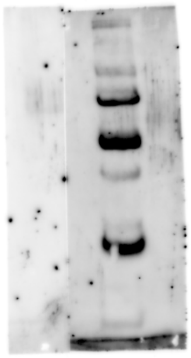

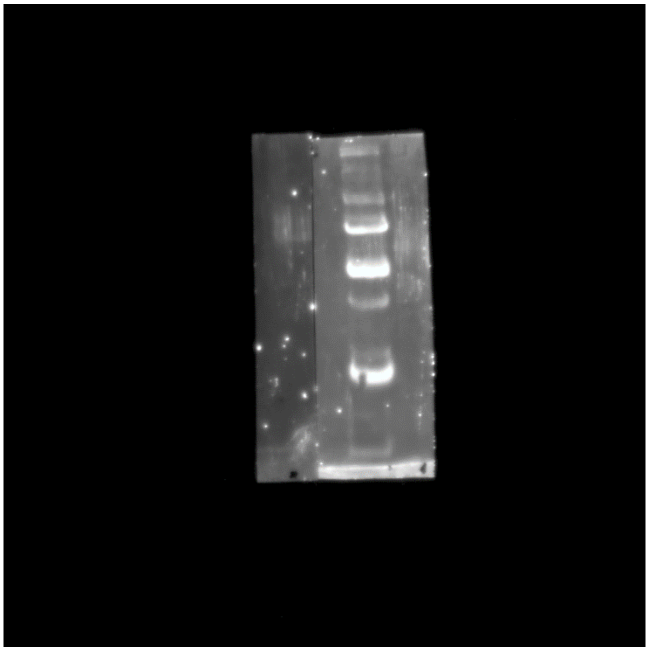

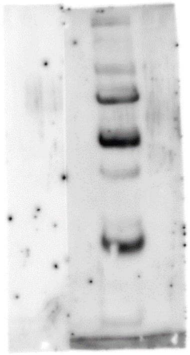

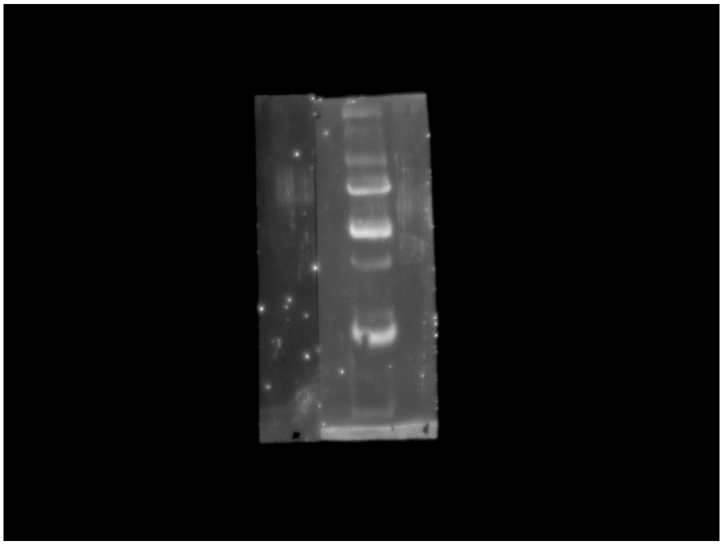

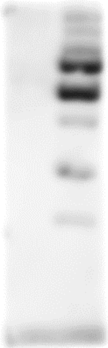

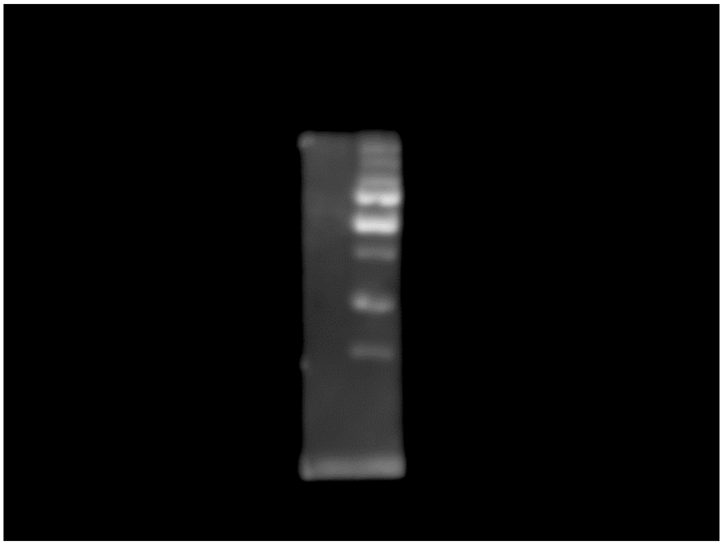

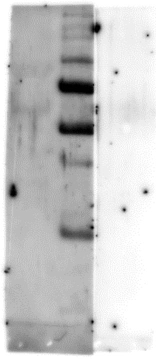

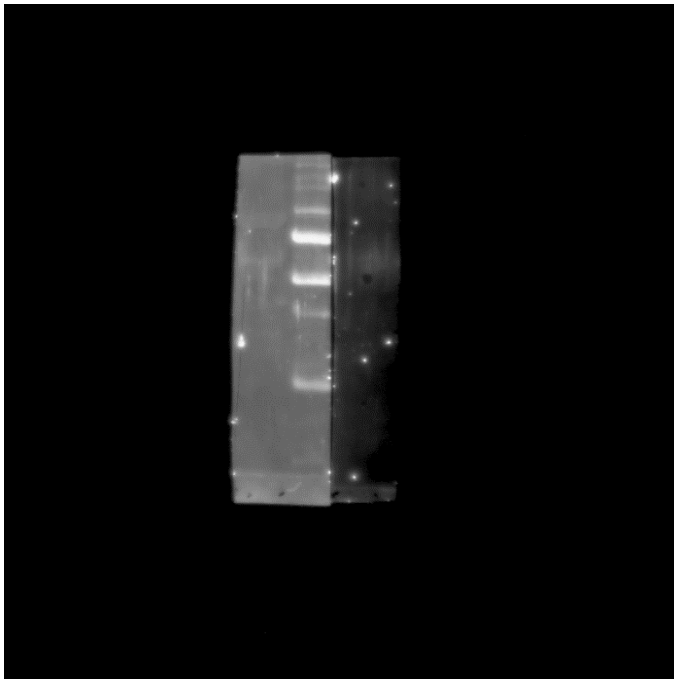

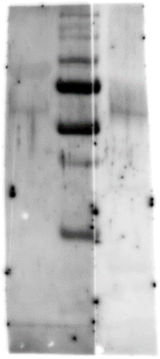

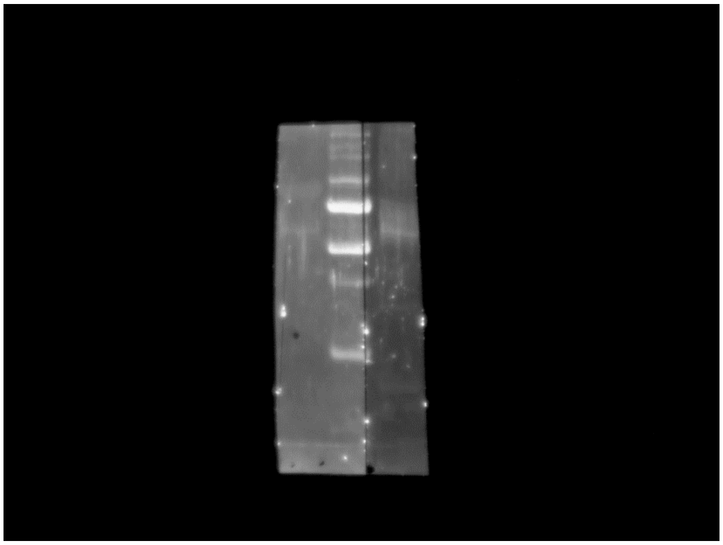

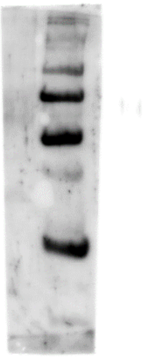

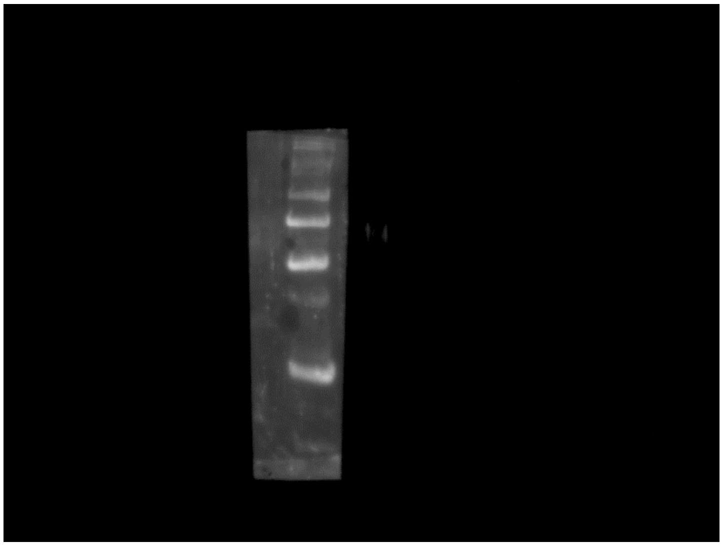

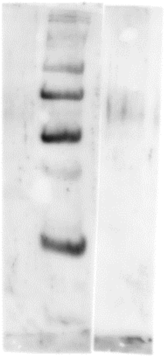

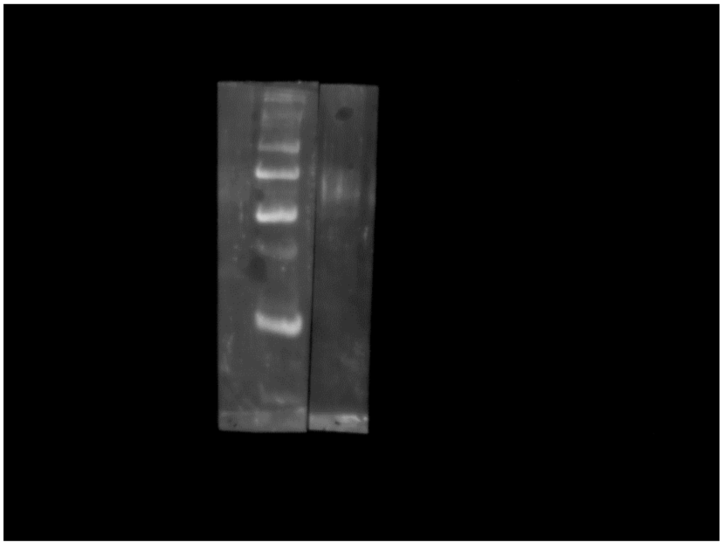

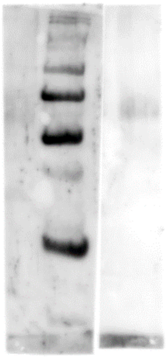

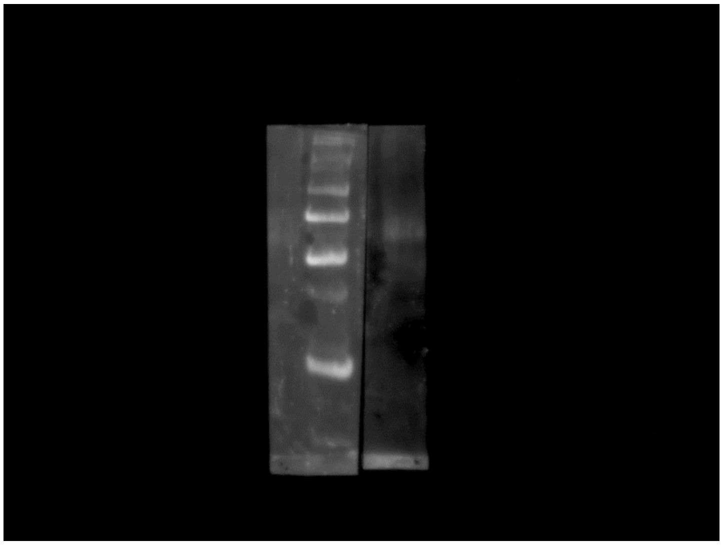

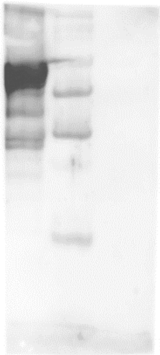

3- Group C

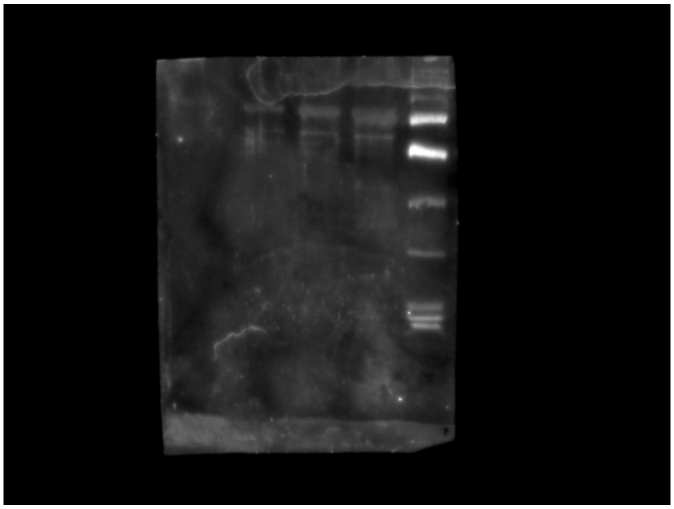

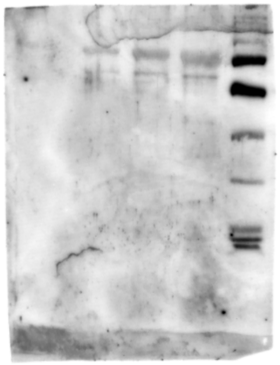

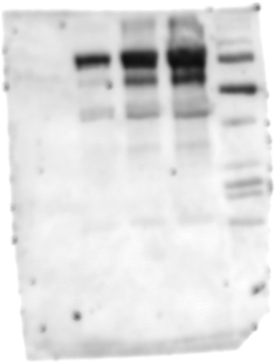

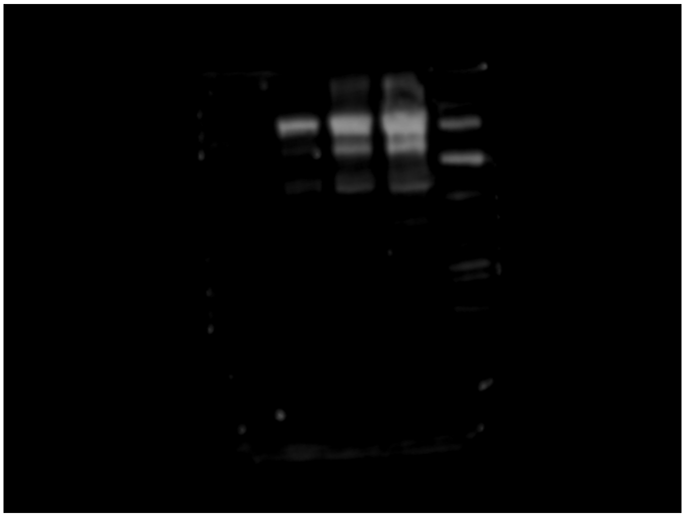

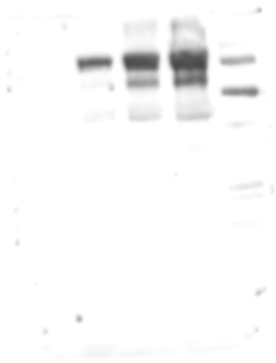

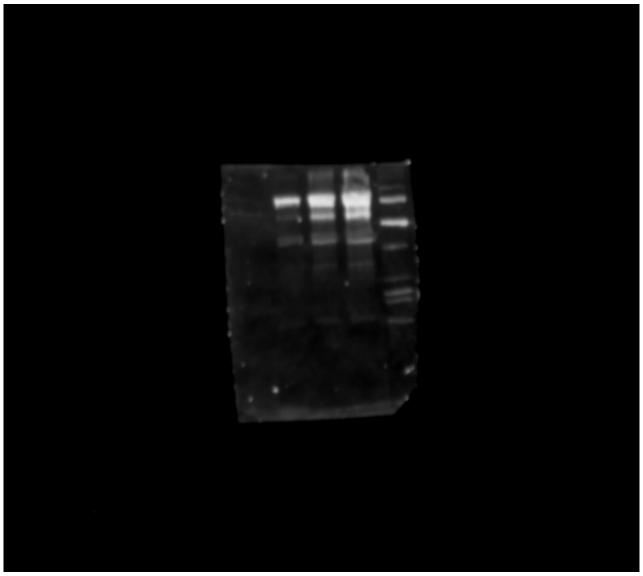

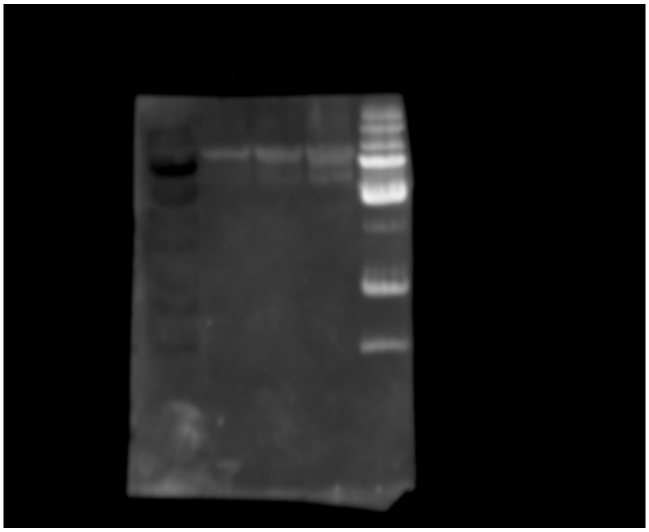

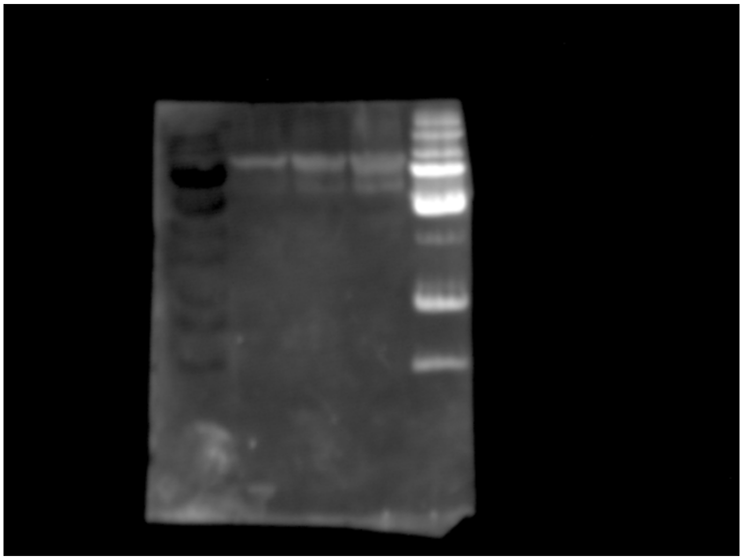

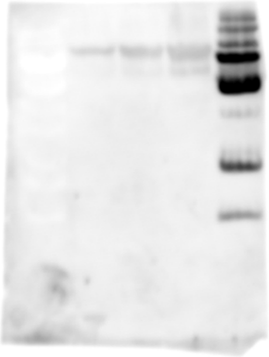

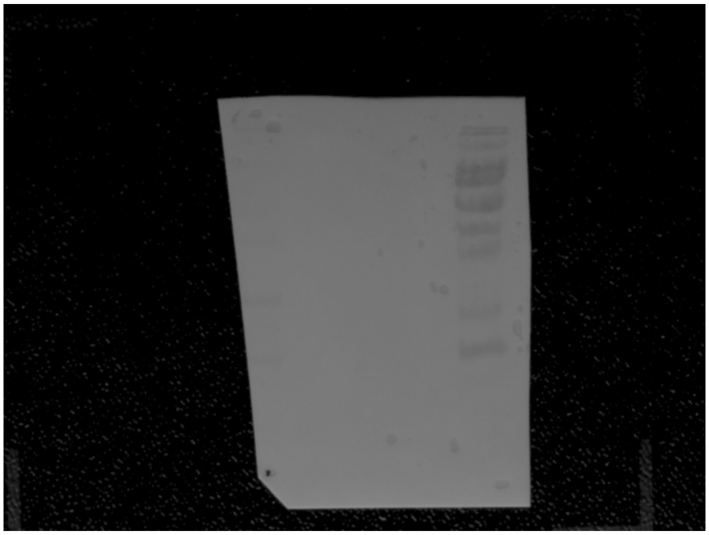

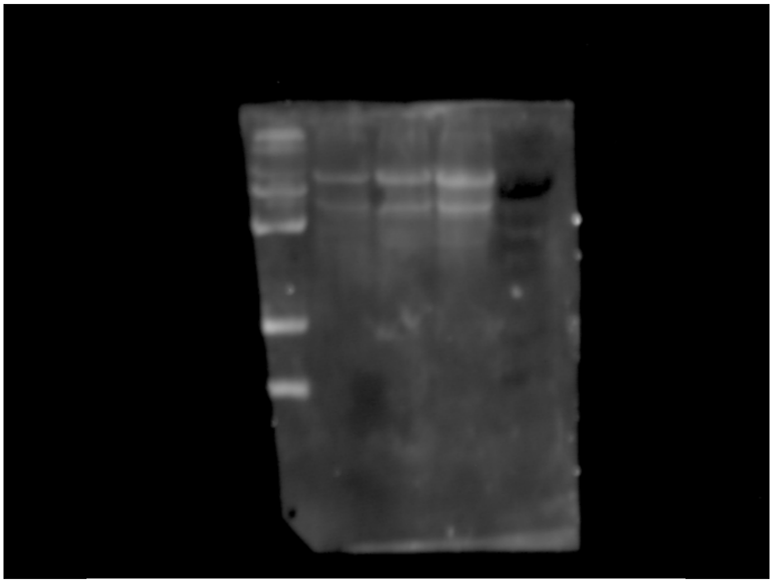

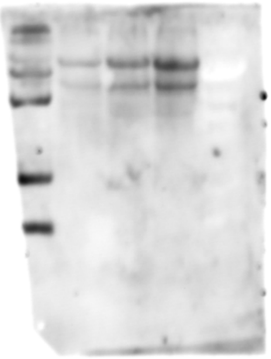

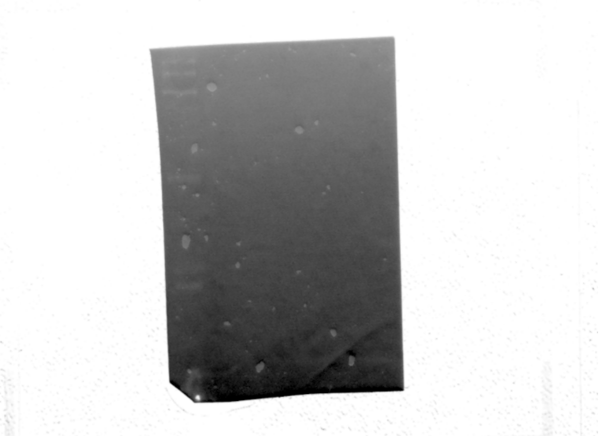

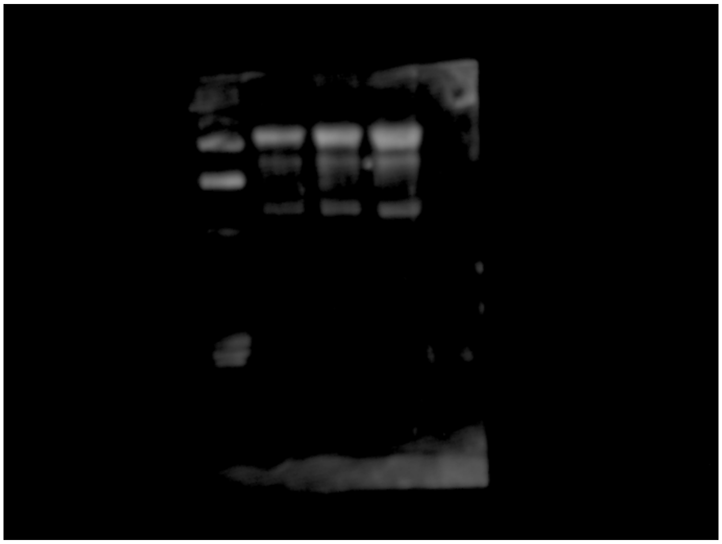

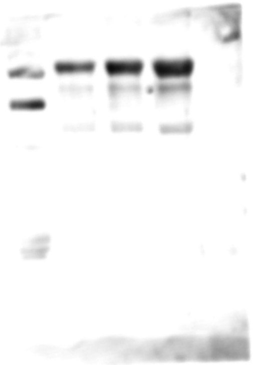

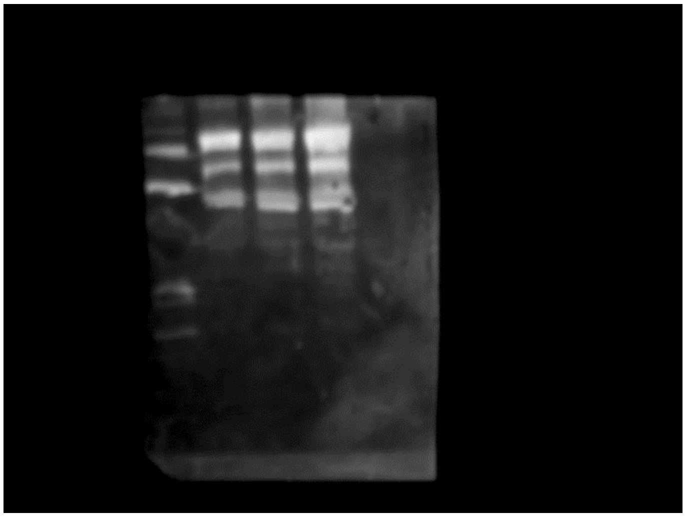

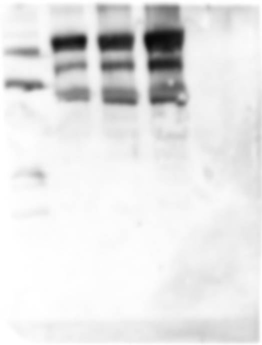

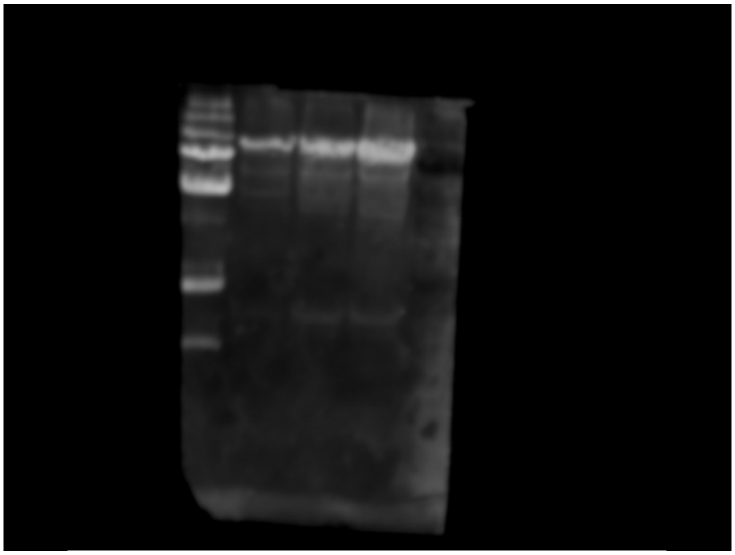

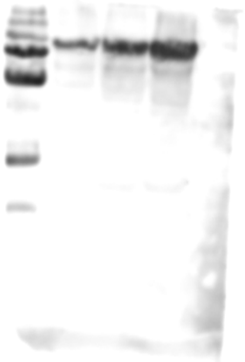

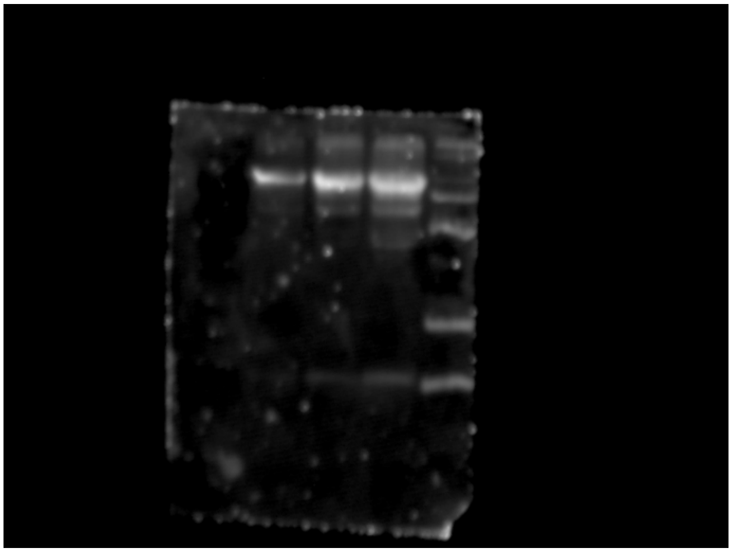

4- Group D

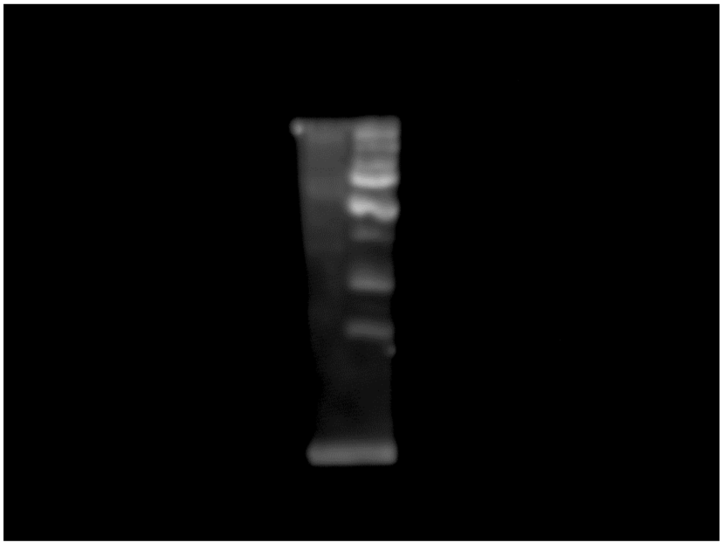

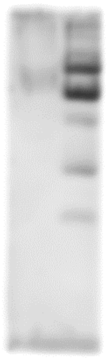

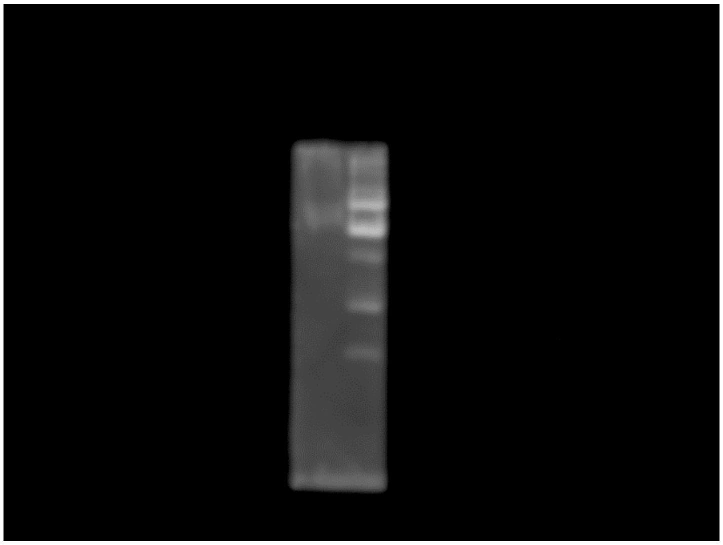
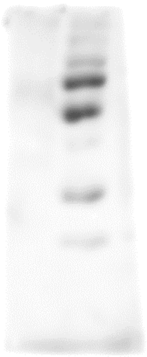
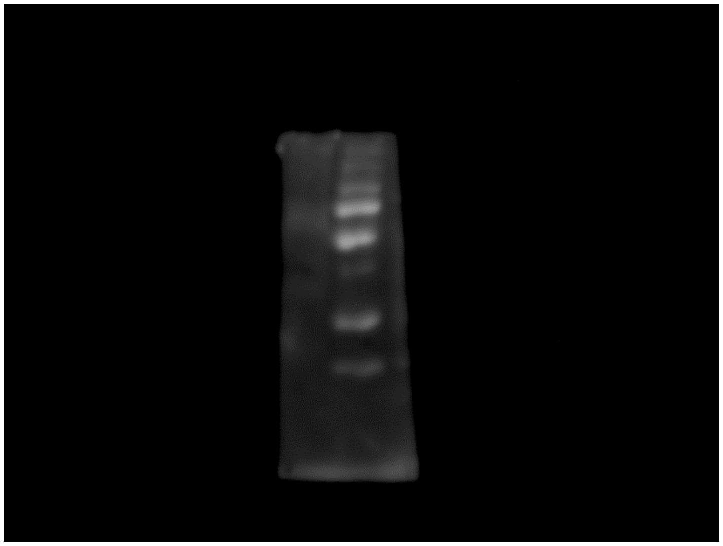

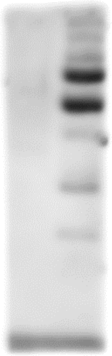
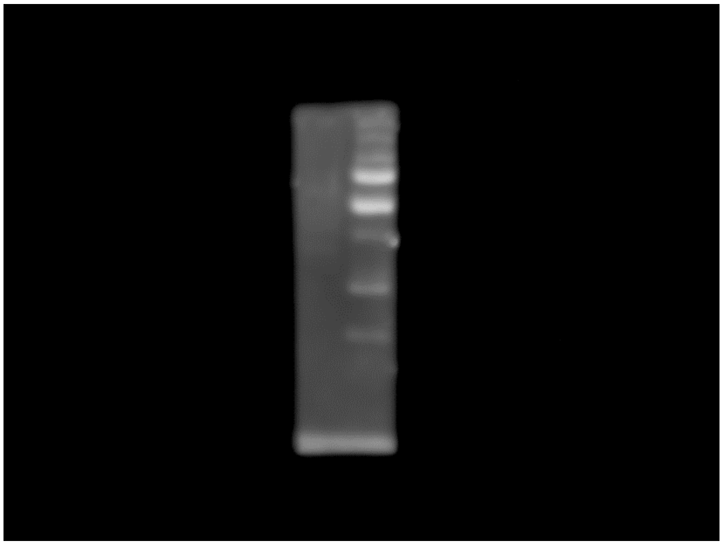
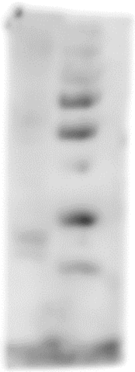

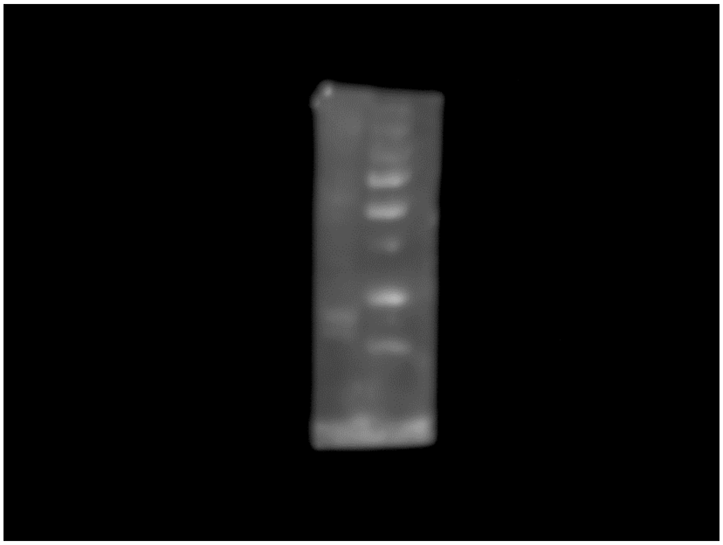

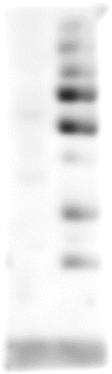

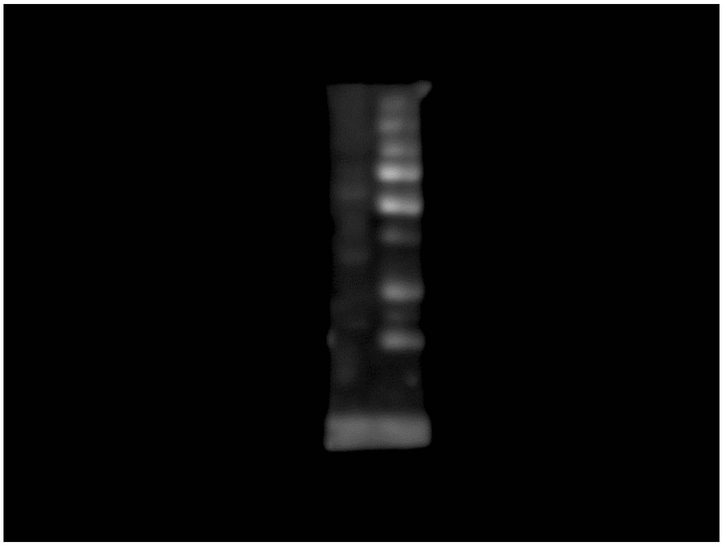

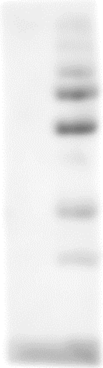

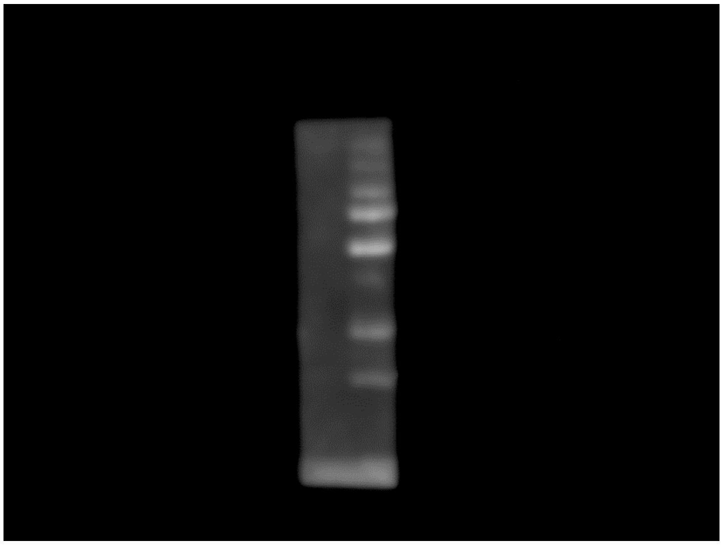

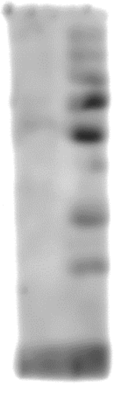

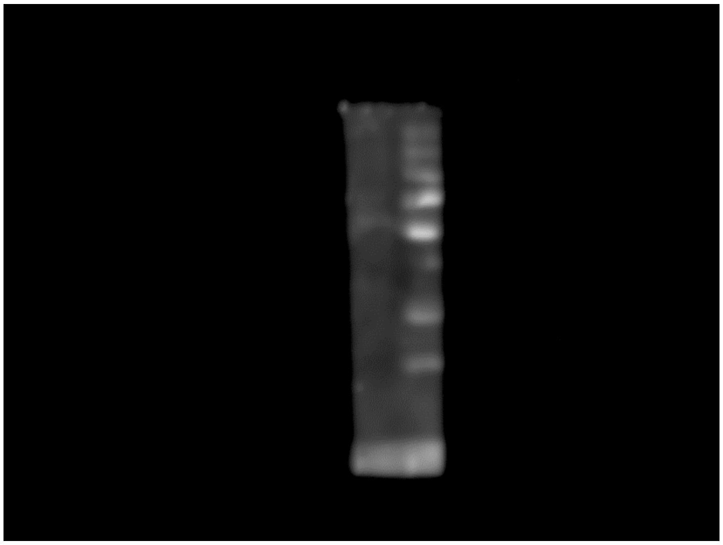

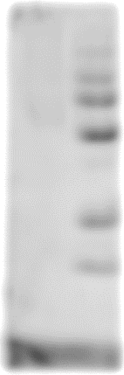

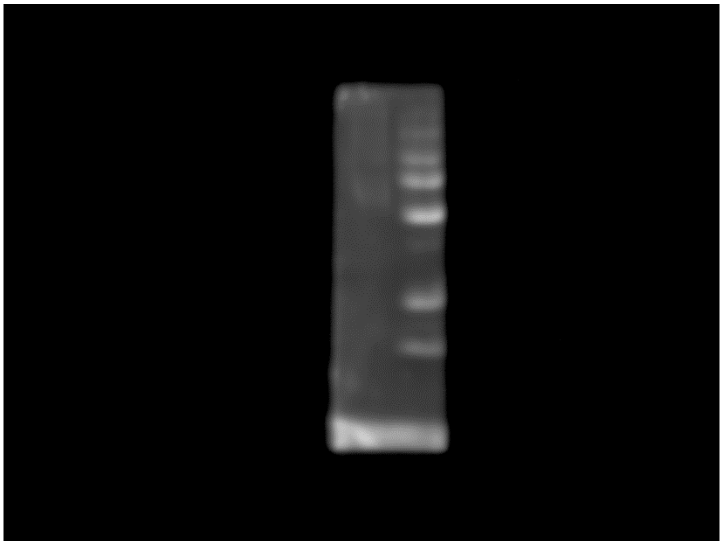

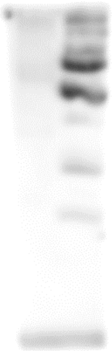

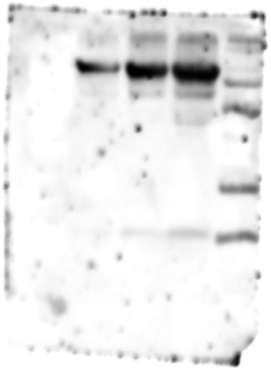

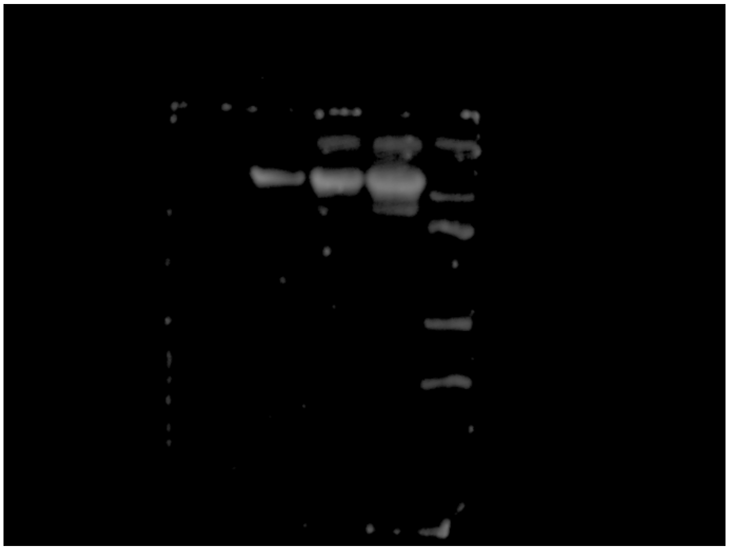

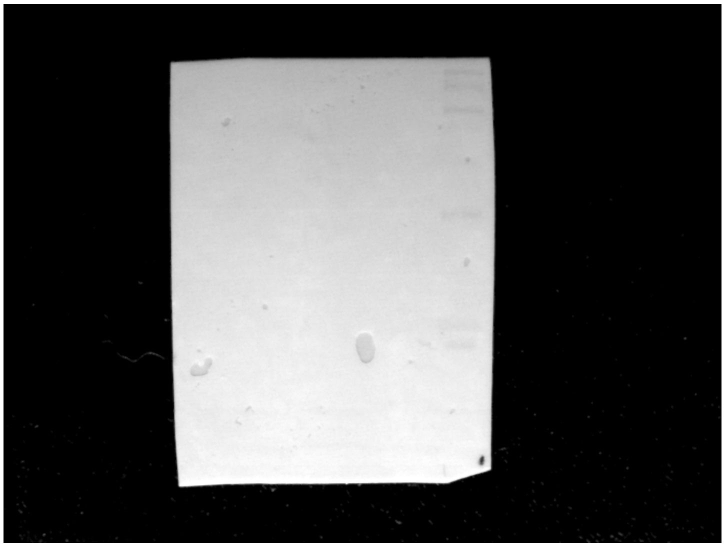

Figure S3. Immunoblots of the OMPs extract of C. jejuni probed with hens' sera. OMPs were prepared from C. jejuni (20 μg per well), separated by SDS-PAGE (S), and electroblotted onto nitrocellulose membranes. The breeders were vaccinated with either 200 μg of C. jejuni OMPs (group A), or 50 μg of CpG ODN (group B), or their combination (200 μg of C. jejuni OMPs and 50 μg of CpG ODN) (group C), or PBS (group D). At four weeks post-primary vaccination, breeders were bled, and 9 serum samples per group were separated and probed on the strips A1-A9, B1-B9, C1-C9, and D1-D9. An empty well (no OMPs loaded) served as a control

Offspring (group A)

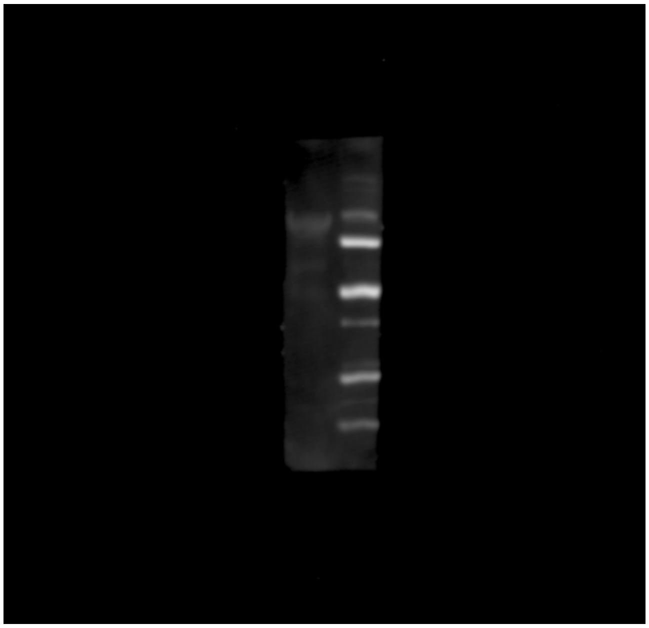

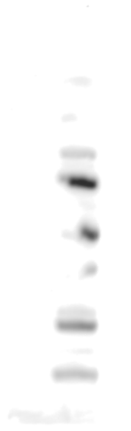

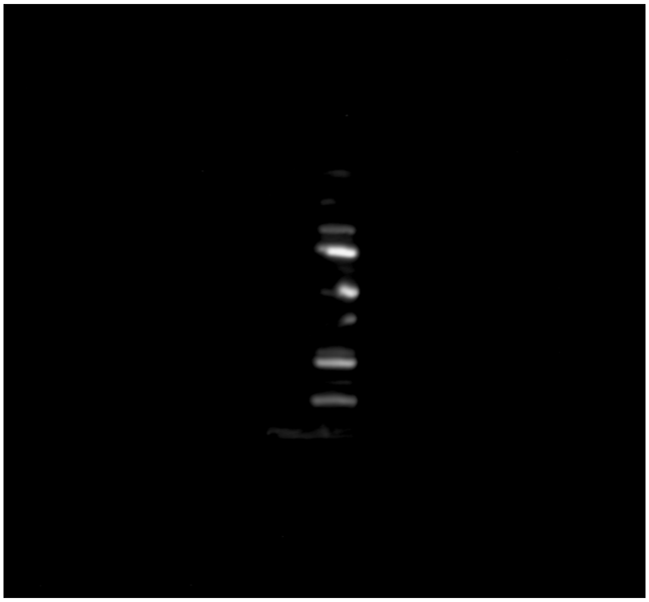

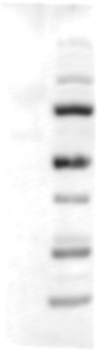

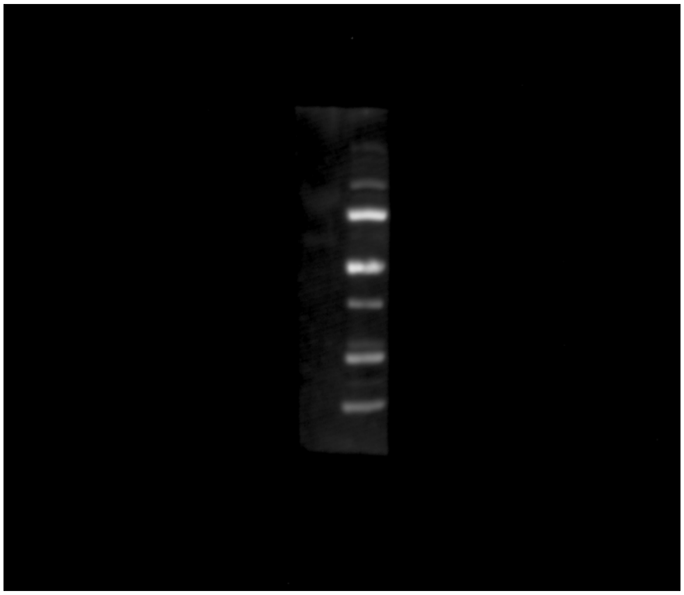

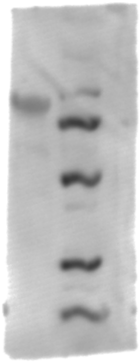

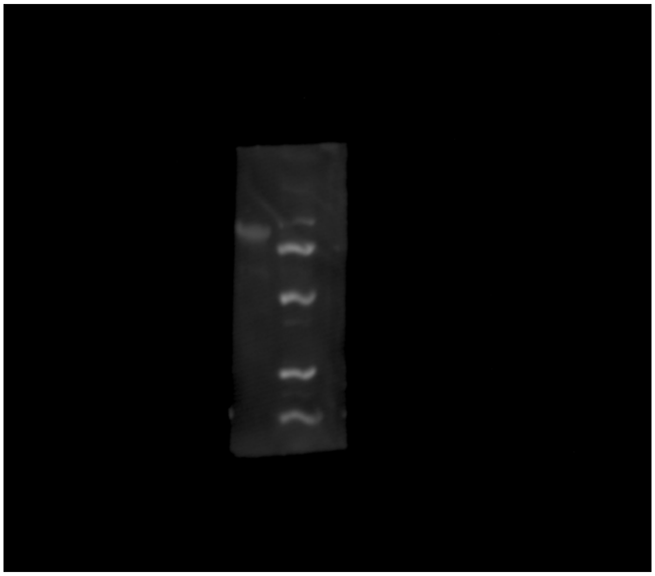

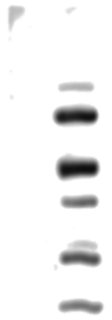

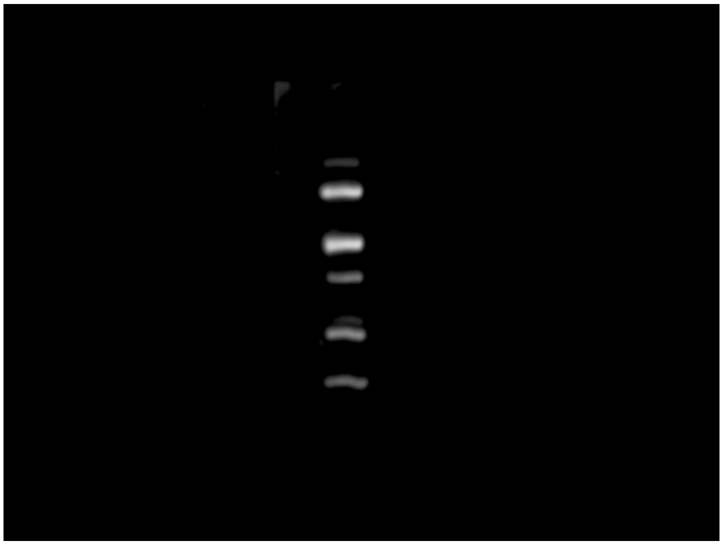

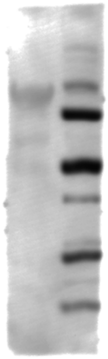

2- Offspring Group B

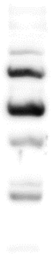

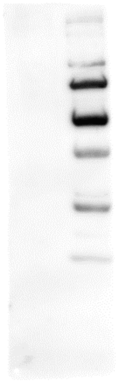

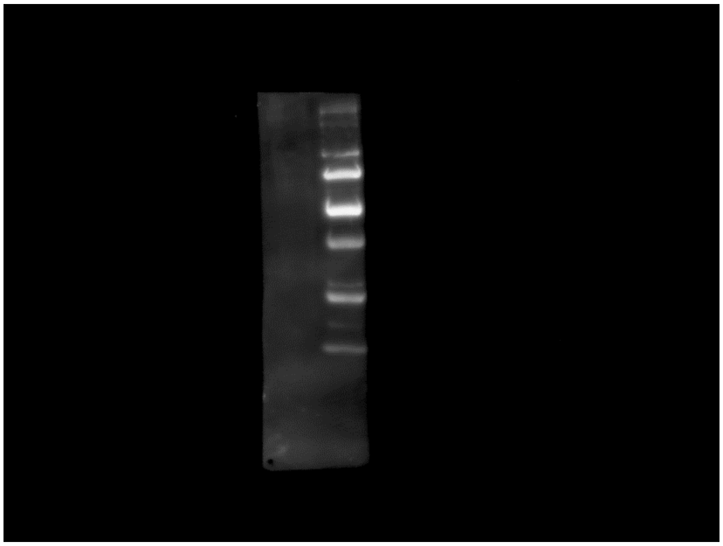

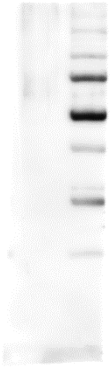

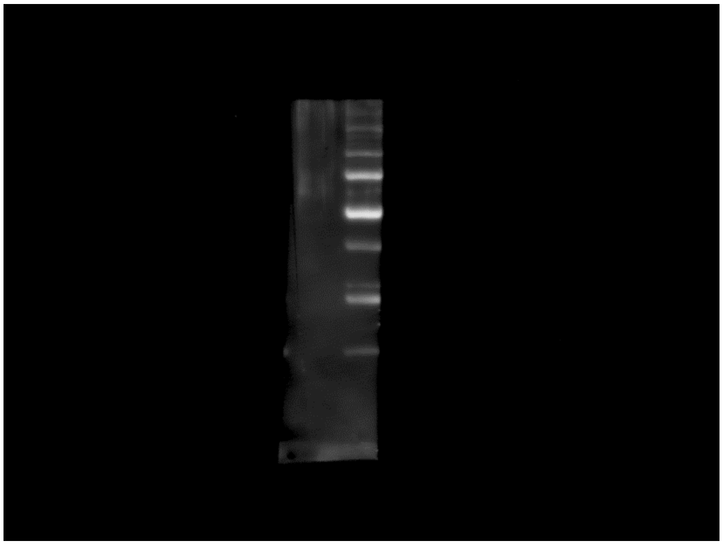

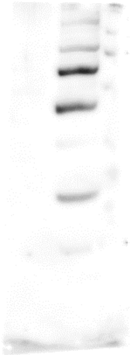

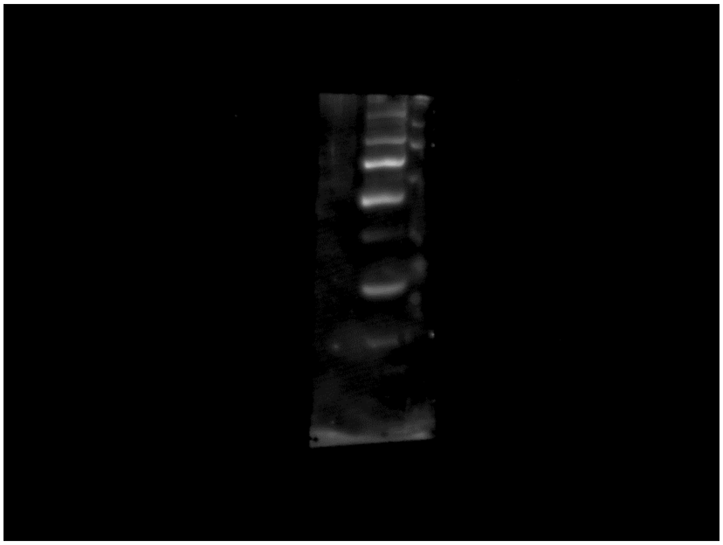

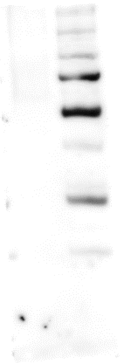

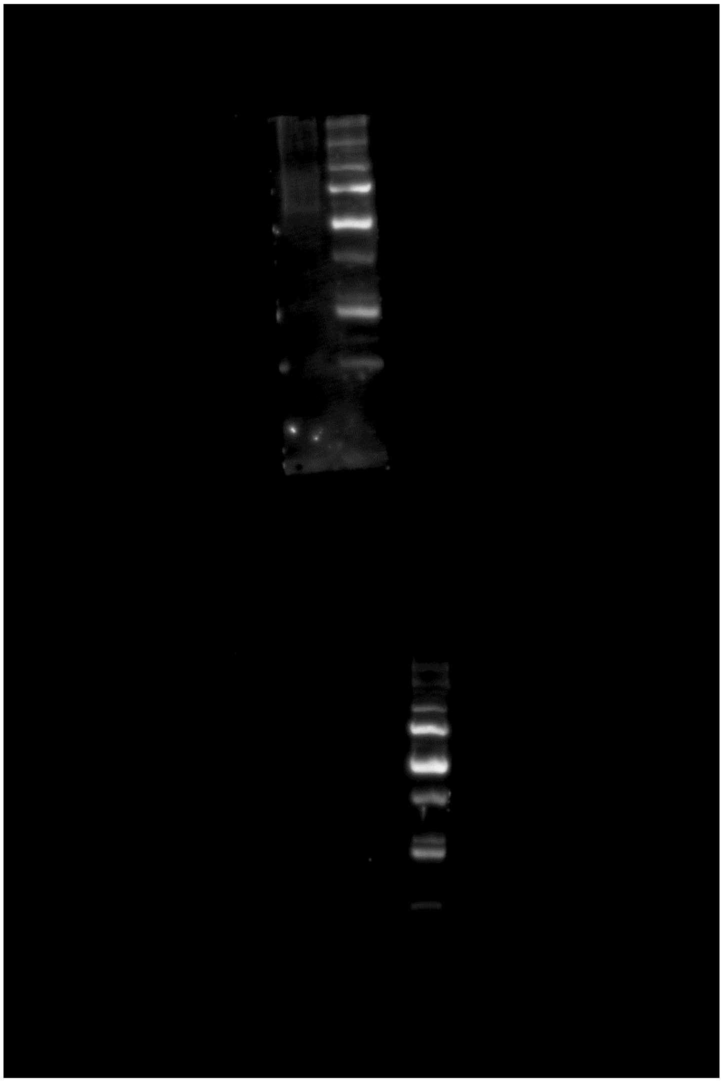

3-Offspring Group C

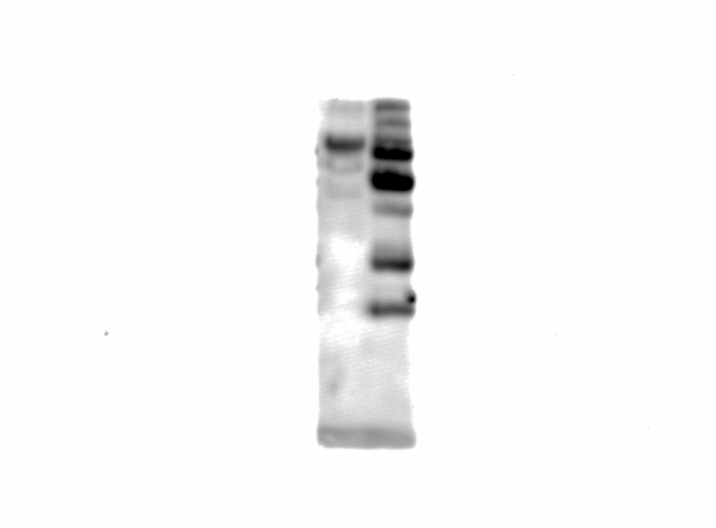

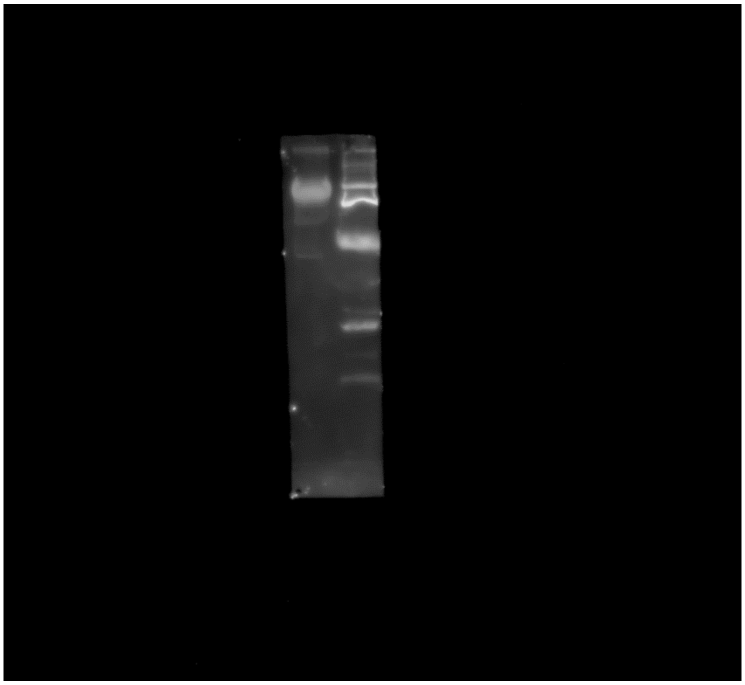

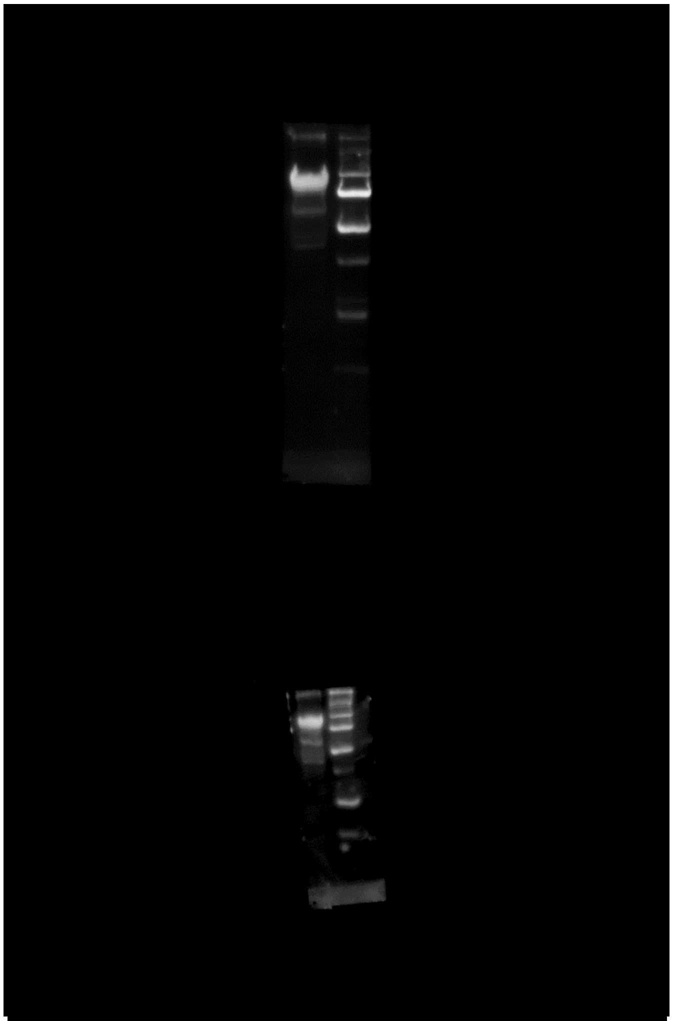

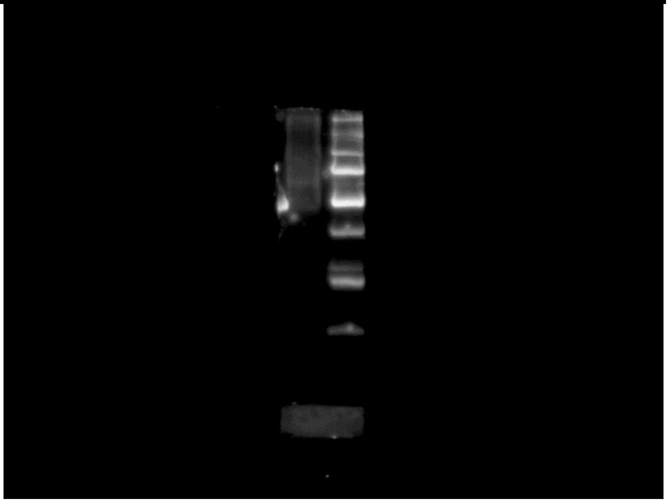

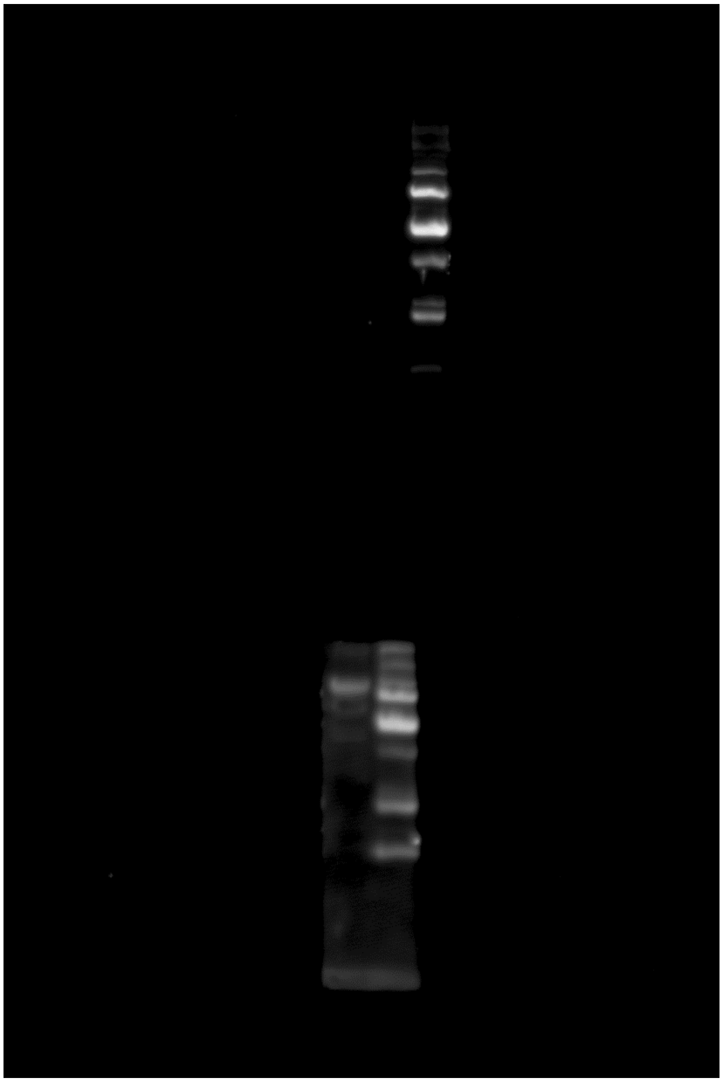

4-Offspring Group D

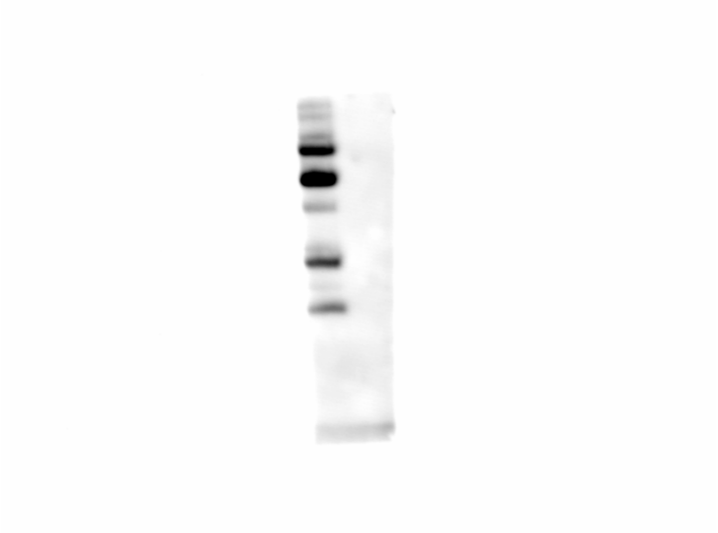
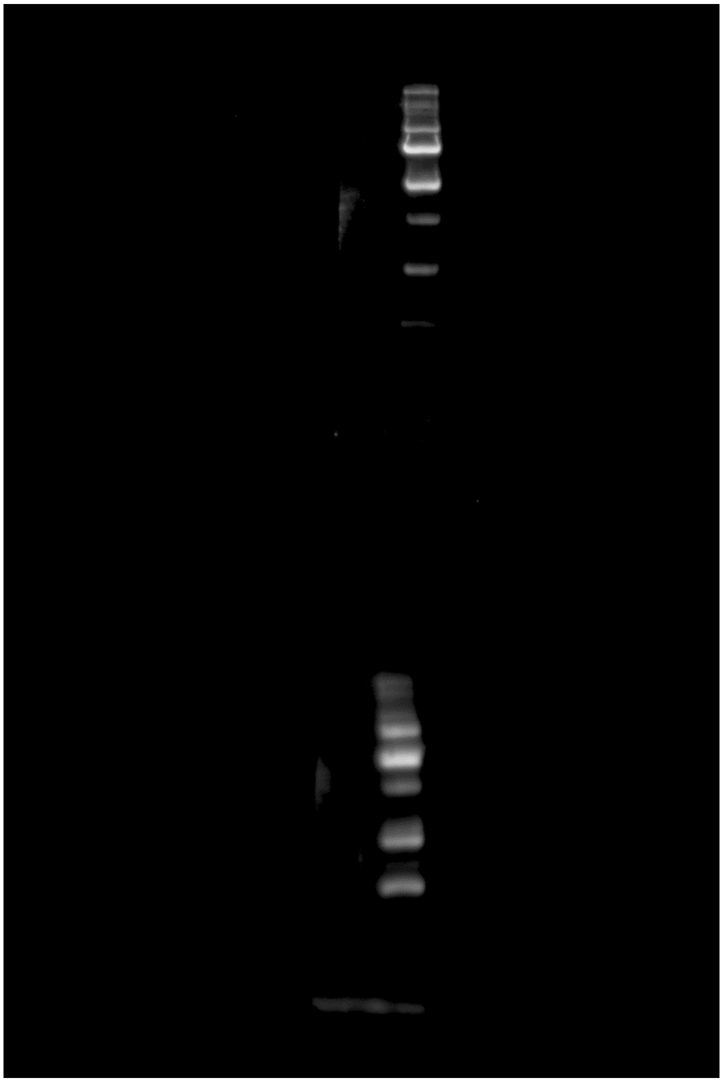

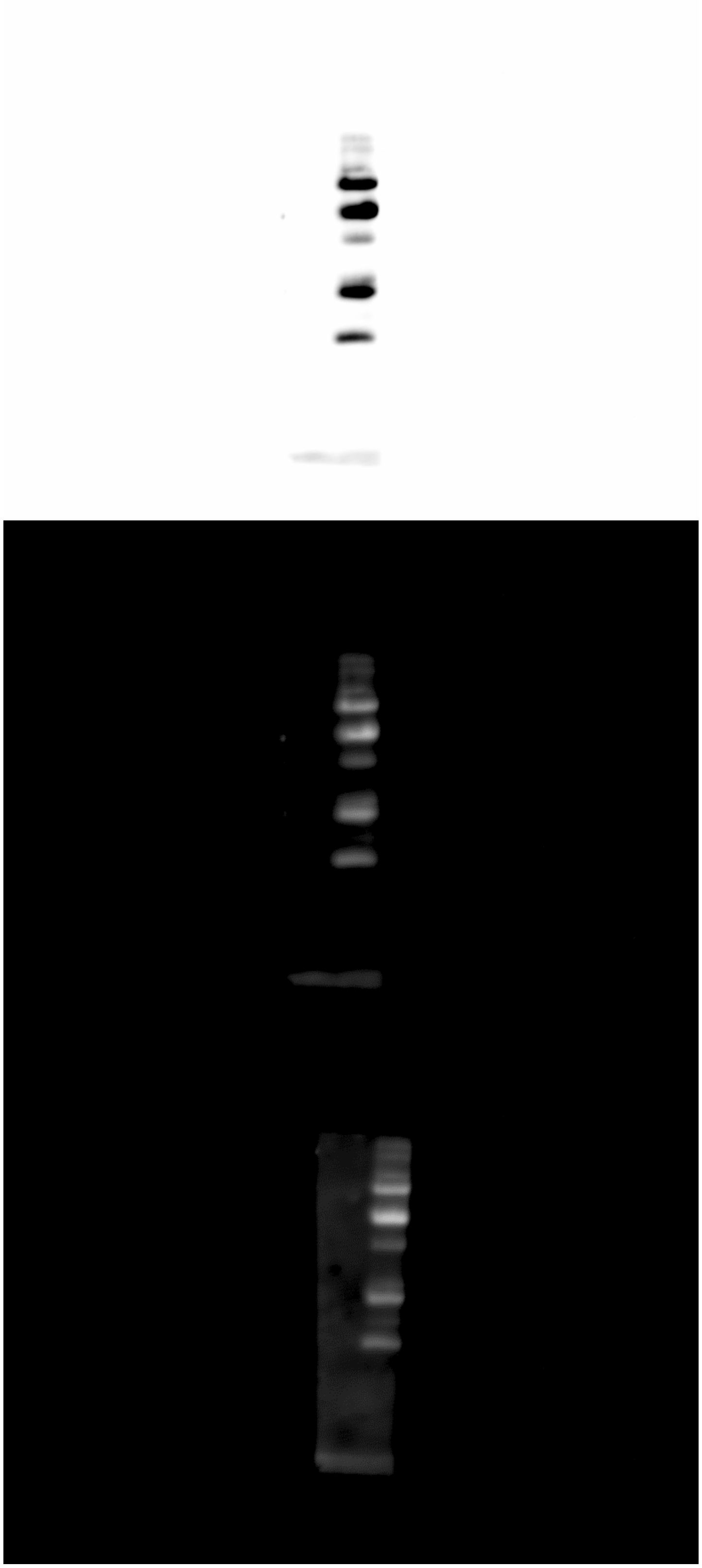

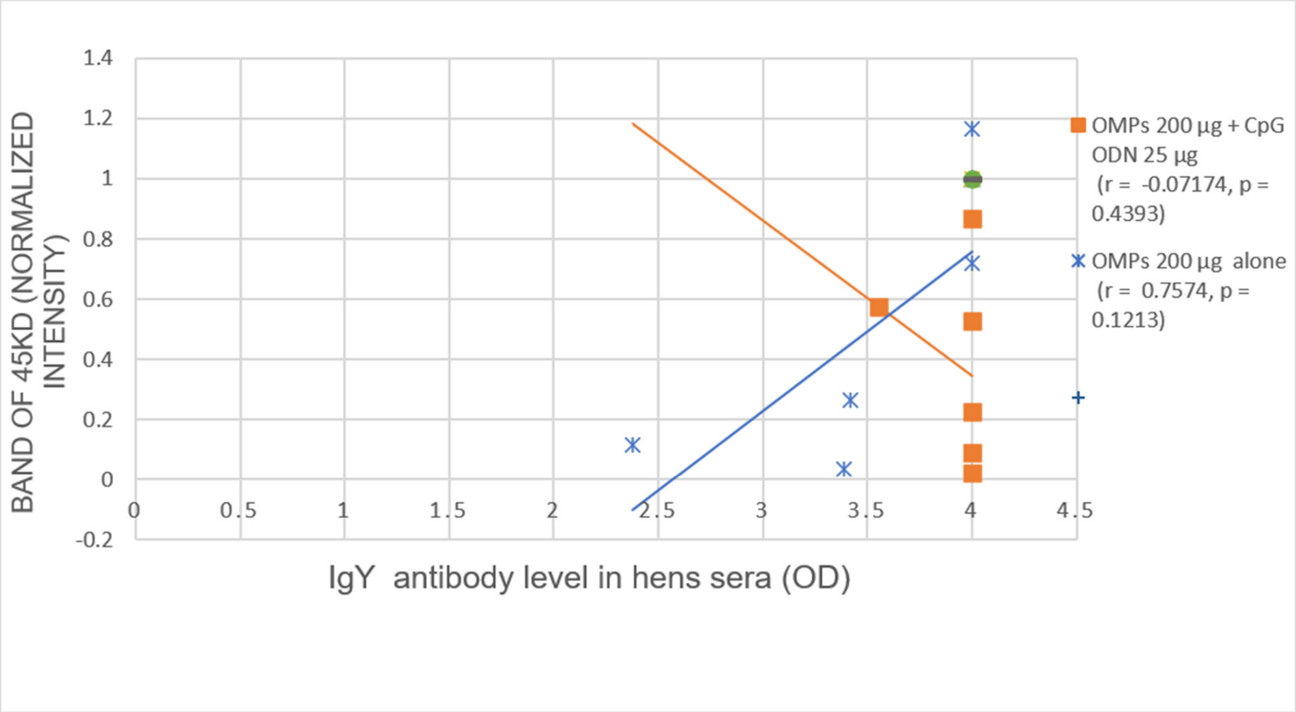

Figure S4. The correlation between the intensity of the reactive 45-kDa band and the IgY antibody levels in the sera of vaccinated hens was determined using Pearson's r correlation coefficient. No correlation was detected (P>0.05).

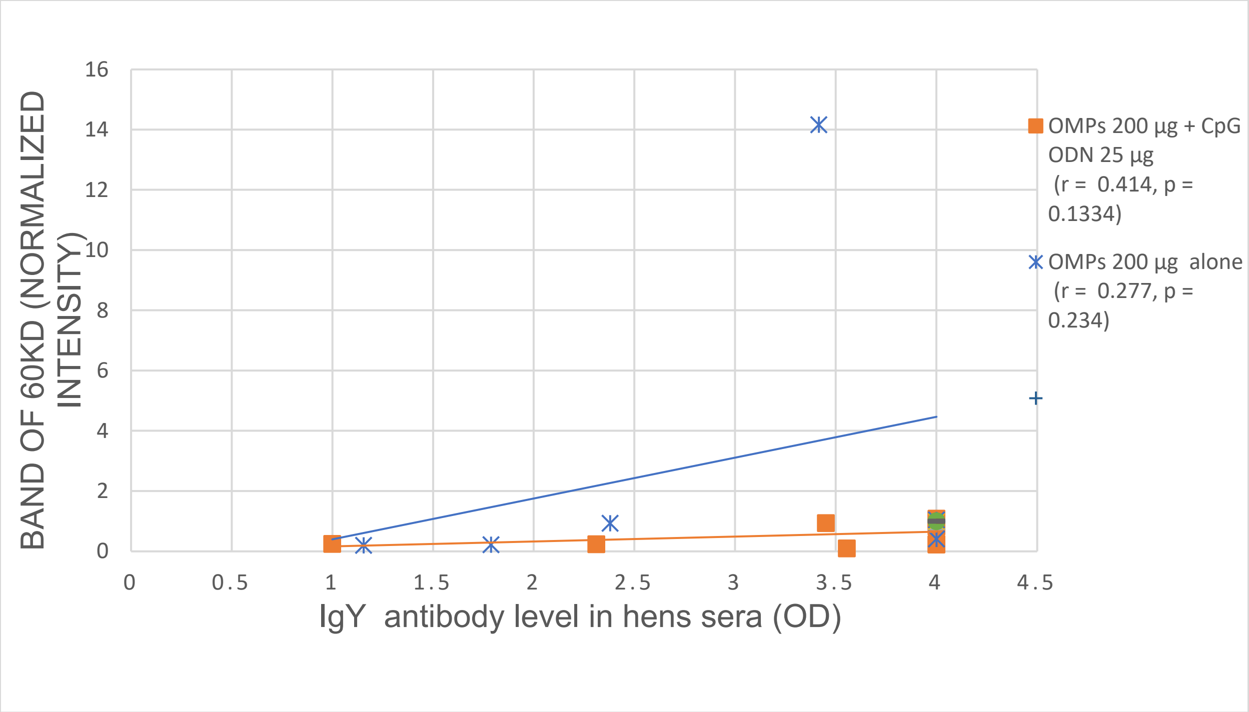

Figure S5. The correlation between the intensity of the reactive 60-kDa band and the IgY antibody levels in the sera of vaccinated hens was determined using Pearson's r correlation coefficient. No correlation was detected (P>0.05).

Supplemental Tables:

Table S2. Characterization of immunogenic, membrane-associated proteins detected in reactive bands with sera from vaccinated hens and their offspring
, identified by UHPLC–MS/MS and analyzed using the UniProtKB database against the C. jejuni 81-176 strain.

No.	MW [kDa]	Protein	Protein Description	Coverage [%]	Score Sequest HT	PSMs
1	42.8	VirB10	Type IV secretion system protein	23	21.43	7
2	43.3	Uncharacterized protein	Uncharacterized protein	3	2.24	1
3	45.7	MOMP/Por A	Major outer membrane protein	53	143.79	35
4	46.1	FlpA	Fibronectin type III domain protein	11	13.32	4
5	49.3	Domain of unknown function (DUF)	DUF6844 domain-containing protein	41	100.63	29
6	59.5	Flagellin	Flagellin	64	2007.09	541
7	59.7	FlaA	Flagellin	58	1733.5	459
8	60.7	KpsD	Capsular polysaccharide ABC transporter	28	42.76	19
9	67.1	FliD	Flagellar hook-associated protein 1	46	73.56	27

10	69.7	FliD	Flagellar hook-associated protein 2	33	97.34	29
11	89.4	FlgE	Flagellar hook protein	31	94.41	25
12	90.9	PflA	Paralyzed flagella protein	5	6.34	3

a percentage of the protein sequences covered by identified peptides.

b Protein score, which is the sum of the scores of the individual peptides.

c Number of identified peptide spectrum matches identified from all included searches.

Table S3. The abundance of the reactive proteins of the C. jejuni-OMPs extract with the sera of vaccinated hens and their offspring
, identified by UHPLC-MS/MS and analyzed by Uniprot KB database against the C. jejuni 81-176 strain.

No.	Protein	Protein Description	Abundance			
			Band1	Band2	Band3	Band4
1	VirB10	Type IV secretion system protein			4.81E+03	4.69E+05
2	Uncharacterized protein	Uncharacterized protein				3.11E+04
3	MOMP/Por A	Major outer membrane protein	1.49E+05	5.14E+04	6.23E+04	3.36E+07
4	FlpA	Fibronectin type III domain protein				2.50E+05
5	Domain of unknown function (DUF)	DUF6844 domain-containing protein	1.68E+05	1.91E+05	2.31E+05	1.08E+07
6	Flagellin	Flagellin	2.92E+08	2.38E+09	4.02E+08	2.69E+07
7	FlaA	Flagellin	1.06E+07	3.69E+07	4.28E+06	6.82E+05
8	KpsD	Capsular polysaccharide ABC transporter	1.94E+05	2.94E+06	1.45E+05	8.44E+03
9	FliD	Flagellar hook-associated protein	6.89E+04	1.99E+05	9.00E+05	7.81E+03
10	FliD	Flagellar hook-associated protein	9.52E+05	1.37E+06	3.76E+05	1.41E+05
11	FlgE	Flagellar hook protein	5.39E+06	9.32E+04	7.33E+04	8.21E+04
12	PflA	Paralyzed flagella protein	4.33E+04			
